# Humanized Mice Reveal New Insights Into the Thymic Selection of Human Autoreactive CD8^+^ T Cells

**DOI:** 10.3389/fimmu.2019.00063

**Published:** 2019-02-04

**Authors:** Yang Li, Nato Teteloshvili, Shulian Tan, Samhita Rao, Arnold Han, Yong-Guang Yang, Rémi J. Creusot

**Affiliations:** ^1^The First Hospital of Jilin University, Changchun, China; ^2^International Center of Future Science, Jilin University, Changchun, China; ^3^Columbia Center for Translational Immunology and Department of Medicine, Columbia University Medical Center, New York, NY, United States; ^4^Naomi Berrie Diabetes Center, Columbia University Medical Center, New York, NY, United States; ^5^Department of Microbiology and Immunology, Columbia University Medical Center, New York, NY, United States

**Keywords:** human thymocyte, thymic selection, clonal deletion, autoreactive, T cell receptor, humanized mouse, antigen-presenting cell, autoantigen

## Abstract

Thymic selection constitutes the first checkpoint in T-cell development to purge autoreactive T cells. Most of our understanding of this process comes from animal models because of the challenges of studying thymopoiesis and how T cell receptor (TCR) specificity impacts thymocyte phenotype in humans. We developed a humanized mouse model involving the introduction of autoreactive TCRs and cognate autoantigens that enables the analysis of selection of human T cells in human thymic tissue *in vivo*. Here, we describe the thymic development of MART1-specific autoreactive CD8^+^ T cells that normally escape deletion and how their phenotype and survival are affected by introduction of the missing epitope in the hematopoietic lineage. Expression of the epitope in a fraction of hematopoietic cells, including all major types of antigen-presenting cells (APCs), led to profound yet incomplete deletion of these T cells. Upregulation of PD-1 upon antigen encounter occurred through the different stages of thymocyte development. PD-1 and CCR7 expression were mutually exclusive in both transgenic and non-transgenic thymocytes, challenging the view that CCR7 is necessary for negative selection in humans. In the presence of antigen, MART1-reactive T cells down-regulated TCR, CD3, CD8, and CD4 in the thymus and periphery. Moreover, expression of secondary TCRs influences MHC class I-restricted T cells to develop as CD4^+^, particularly regulatory T cells. This new model constitutes a valuable tool to better understand the development of autoreactive T cells identified in different human autoimmune diseases and the role of different APC subsets in their selection.

## Introduction

T cells acquire a T cell receptor (TCR) during their development in the thymus, and then become exposed to self-MHC for positive selection, and to MHC/self-peptide complexes for negative selection, leading to a purge of most autoreactive T cells before the remaining mature T cells can be released into the periphery (1, 2). Most of what we know regarding mechanisms of thymic selection comes from animal models. Some studies performed on human thymic tissue, usually removed during cardiac surgery, have offered snapshots on the phenotype and distribution of human thymocytes, with limited insights on their function. However, longitudinal studies of human immune cells and tissues are not feasible beyond blood samples, and using human thymic tissue to assess the development and fate of thymocytes in relation to their antigen specificity is challenging. It is now possible to generate multiple “identical” humanized mice from a single human donor that allow longitudinal studies as well as various manipulations such as introduction of specific TCRs and/or antigens into the hematopoietic stem cells (HSCs) that will reconstitute the human immune system.

A few studies have assessed the expression of a number of self-antigens by human thymic antigen-presenting cells (APCs) for thymic selection, confirming medullary thymic epithelial cells (mTECs) as the primary source of self-antigens (3–5). However, not all tested antigens were found to be expressed, and those that were detected were highly variable in expression (4, 6). Moreover, expression of a particular autoantigen does not guarantee that all epitopes will be presented to thymocytes. A good example is the melanocyte antigen MART1 (encoded by the *MLANA* gene). Despite expression of MART1 in human mTECs (3, 4), alternative splicing generates products that lack approximately 1/3 of the protein on the N-terminal side (including the HLA-A2-restricted MART1_26−35_ epitope) (7). As a result, T cells specific for the MART1_26−35_ epitope are not properly deleted in the thymus and accumulate in the periphery (8). These T cells acquire an anergic rather than naïve phenotype (9), suggesting that they may encounter their antigen in the periphery, possibly in the skin-draining lymph nodes. As MART1 is also a prominent melanoma antigen, T cells specific for this antigen have been cloned (10) and their TCRs transduced into mature polyclonal T cells for adoptive T cell immunotherapy of melanoma (11, 12). However, in this case, TCR transduction can engender unwanted pairings between transgenic (Tg) and endogenous TCR chains, decreasing the amount of desired TCR on surface and increasing the chance of off-target specificity. Alternatively, such MART1-reactive T cells have been produced in humanized mice from TCR-transduced HSCs developing in a HLA-A2 Tg mouse thymus (13) or a grafted HLA-A2 human thymus (14–16), which prevented expression of the endogenous TCRβ chain (13, 15).

We capitalized on such humanized mouse models and on the fact that MART1-reactive CD8^+^ T cells escape thymic deletion to devise a system wherein the missing epitope is re-introduced in the system with the goal of modeling thymic selection of those high-avidity autoreactive T cells in the human thymus. In the present study, we have expressed a strong T cell epitope in some of the HSCs used to reconstitute humanized mice. We show that the HSCs can give rise to all major types of hematopoietic APCs, which can be found both in the human thymus and in peripheral lymphoid tissues of the mouse. In the presence of peptide-expressing APCs in the thymus, nearly all specific TCR-expressing T cells upregulate PD-1 instead of CCR7 as they undergo deletion. In absence of antigen, we observed that the TCR-expressing cells develop primarily as naïve CD8^+^ T cells, but that high level of Tg-TCR expression in conjunction with more frequent and higher expression of endogenous TCRα chains generate secondary TCRs that contribute to the development of some of the Tg-TCR^+^ T cells as CD4^+^ T cells, including regulatory T cells (Tregs). This *in vivo* model opens new possibilities for studying the thymic development of human autoreactive T cells, the contribution of specific subsets of APCs to central tolerance and the implications of dual TCR expression in autoimmunity and tumor immunity.

## Materials and Methods

### Mice, Human Tissues, and Cells

NSG mice (NOD.Cg-Prkdc^scid^ Il2rg^tm1Wjl^/SzJ; stock 005557) were obtained from the Jackson Laboratory. They were bred in our maximal barrier (Helicobacter and Pasteurella-free, specific pathogen-free) facility and both males and females were used between 6 and 8 weeks of age. Human fetal thymus and liver tissues of gestational age of 17–20 weeks were obtained from Advanced Bioscience Resource (Alameda, CA). The thymic tissue was cut into small fragments approximately 1 mm^3^ in size; and human CD34^+^ fetal liver cells (FLCs) were purified by magnetic-activated cell sorting using anti-human CD34 microbeads (Miltenyi Biotech, Aubum, CA). The prepared human thymic tissue fragments and CD34^+^ FLCs were then cryopreserved in liquid nitrogen until use. Melanoma cell lines Mel-A375 and Mel-624 were obtained from Dr. Steven A. Rosenberg. Protocols involving the use of discarded human tissues and animals were approved by the Institutional Review Board of the Human Research Protection Office and the Institutional Animal Care and Use Committee at Columbia University.

### Lentiviral Constructs (TCR, Antigen), Lentivirus Preparation, and HSC Transduction

**Figure 1:**
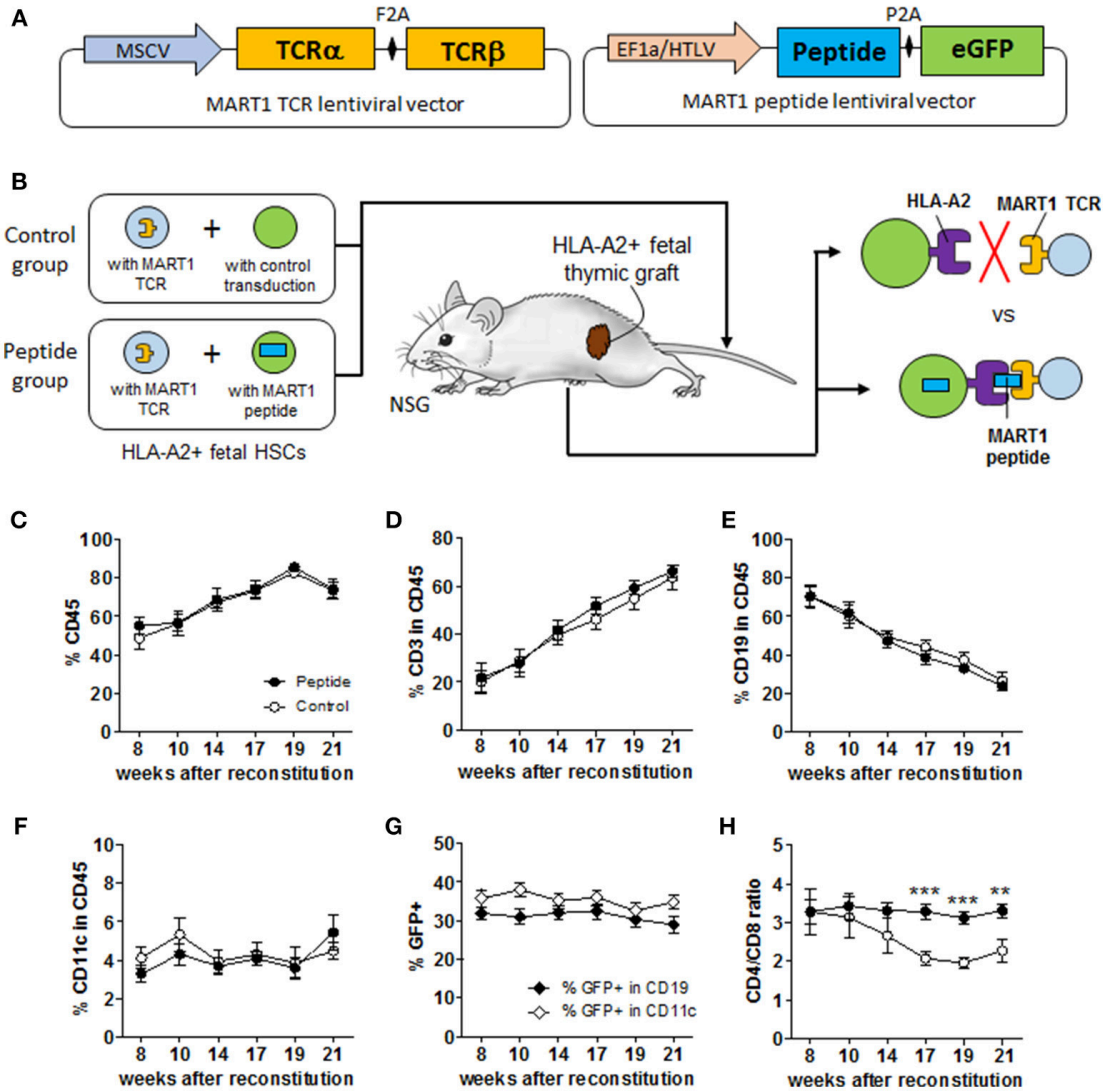
Experimental scheme and immune reconstitution of humanized mice. **(A)** Simplified overview of MART1-TCR and MART1 peptide-expressing lentiviral vectors. **(B)** Experimental scheme of the MART1 model depicting the characteristics and expected outcomes of the Control and Peptide groups. Flow cytometric determination in the blood at different time points after HSC and thymic implantation of: **(C)** % human CD45^+^ cells, **(D)** % CD3^+^ T cells, **(E)** CD19^+^ B cells, and **(F)** CD11c^+^ cells among CD45^+^ cells. **(G)** % of GFP^+^ (MART1 peptide-expressing cells) among CD19^+^ and CD11c^+^ cells measured in the Peptide group. **(H)** D4/CD8 ratio measured from total CD8^+^ and CD4^+^ T cells. All graphs show the mean ± SEM from Control group (open circles, *n* = 9) and Peptide group (closed circles, *n* = 8). Data were pooled from two independent and highly reproducible experiments. No significant differences were seen between the two groups by two-way ANOVA; panel H shows significant differences by *t*-test analysis.

The lentiviral vector expressing the MART1-reactive TCR clone DMF5 has been previously described (16). The two TCR chains were separated by the F2A cleavage site and their expression was driven by the MSCV promoter (Figure 1A). The antigen-expressing vector is a pLVX lentiviral vector modified to co-express the MKELAGIGILTVK peptide and EGFP under control of the EF1α/HTLV promoter (Figure 1A). The construct containing EF1α/HTLV composite promoter, MART1 peptide with KOZAK sequence, P2A cleavage site, EGFP, MND promoter, and mCherry was codon-optimized and synthesized (Genewiz, NY, USA), and introduced into pLVX-EF1a-IRES-mCherry using BstBI and MluI sites. The pLVX vector was also modified to introduce a synthesized truncated Δ3′LTR using KpnI and NheI sites to make the vector self-inactivating. MART1-TCR lentiviral vector was amplified in Mach1-T1 (Invitrogen/ThermoFisher), all other plasmids were amplified in DH5α and isolated using GenElute Endotoxin-free Plasmid Maxiprep kit (Sigma). Lentiviral particles were produced by co-transfection of a 3-plasmid system consisting of the transfer vector (TCR) and packaging plasmids (pVSV-G and pΔ) using CaCl_2_ into 293 T cells in 175 cm^2^ flasks (17). Lentivirus supernatant was collected 48 h post-transfection, concentrated by ultracentrifugation at 22,000 rpm for 2.5 h (Optima XE-90, Beckman Coulter) and stored at −80°C until use. A test aliquot was used for titration of antigen-encoding lentivirus based on GFP expression in 293 T cells and of TCR-encoding lentivirus based on HLA-A^*^0201/MART1 (ELAGIGILTV) Tetramer (MBL Inc.) staining on TCR-deficient Jurkat cells (clone J.RT3-T3.5). Human CD34^+^ HSCs were transduced by lentivirus expressing MART1-TCR, MART1 peptide or mCherry (pLVX-EF1a-IRES-mCherry as control) while stimulated overnight in IMDM medium containing 50 ng/mL rhSCF, 25 ng/mL TPO, 10 ng/mL IL-3 (all from R&D, Minneapolis, MN), and 50 ng/mL Flt-3L (eBioscience, San Diego, CA) in 24-well plates pre-coated with Retronectin (Takara Bio Inc.). Cells were washed twice before injection into recipient mice, a small aliquot of transduced cells were cultured for 3 additional days to validate high transduction efficiency by measuring the fraction of GFP^+^ cells by FACS (Figure S1A).

### Generation of Humanized Mice

NSG mice with or without thymectomy, as well as HLA-A2 Tg mice, were conditioned with sublethal (1.5 Gy) total body irradiation, and injected intravenously with human CD34^+^ HSCs (2 × 10^5^/mouse). Mice were then implanted with a cryopreservation-treated fetal thymus fragment (~1 mm^3^) from the same fetal donor under the recipient kidney capsule, as previously described (18). Mice in Peptide group were injected with a mixture of 1 × 10^5^ MART-1 TCR-expressing HSCs and 1 × 10^5^ MART1 peptide-expressing HSCs, while mice in Control group were injected with a mixture of 1 × 10^5^ MART-1 TCR-expressing HSCs and 1 × 10^5^ control transduced HSCs (Figure 1B). Two independent cohorts, each containing both groups of mice were prepared and yielded remarkably similar results, thus results were pooled. At end point (24–29 weeks after reconstitution), all collected tissues were assessed for size, weight and/or cellularity prior to analysis.

### Tetramer and Antibodies

Levels of human hematopoietic cells and phenotyping of antigen-specific T cells in blood of humanized mice (starting from 8 weeks after reconstitution until end point) and in spleen, lymph nodes, and thymic graft (at end point) were determined by flow cytometric analysis using various combinations of the following monoclonal antibodies: anti-human CD45 (clone HI30), CD19 (clone HIB19), CD3 (clone SK7), CD4 (clone OKT4), CD8 (clone HIT8a), CD25 (clone BC96), CD127 (clone A019D5), FOXP3 (clone 206D), HLA-DR (clone L243), CD1c (clone L161), CD123 (clone 6H6), CD141 (clone M80), CLEC9a (clone 8F9), CCR7 (clone G043H7), and isotype controls were from Biolegend; anti-human CD11c (clone B-ly6), CD14 (clone M5E2), CD45RA (clone HI100), CD45RO (clone UCHL1), PD-1 (clone EH12.1), TIM-3 (clone 7D3), anti-mouse CD45 (clone 30-F11) and Ter119 (clone TER-119), and isotype controls were from BD Biosciences (San Diego, CA). MART1-TCR^+^ T cells were identified with the HLA-A^*^0201/MART1 (ELAGIGILTV) Tetramer. PBMCs were prepared using density gradient centrifugation with Histopaque 1077 (Sigma-Aldrich, St. Louis, MO). Thymocytes were prepared by digestion using 2 mg/mL Collagenase Type II (Gibco/ThermoFisher) and 0.1 mg/mL DNase I (Sigma) for 30 min at 37°C. Analysis was performed on a FACS Fortessa (Becton Dickinson, Mountain View, CA). Dead cells were excluded from the analysis using fixable or non-fixable viability dyes. All samples were collected on BD Fortessa and LSRII flow cytometers.

### Immunofluorescence

Half of the thymic graft from each humanized mouse was fixed in 4% paraformaldehyde for 30 min at room temperature, then transferred to 30% glucose PBS at 4°C overnight. Tissues were then embedded in OCT compound and frozen. Cryosections (5 μm) were blocked with goat serum (Vector Laboratories) for 15 min, and stained with following primary antibodies: rabbit anti-GFP (Millipore/Sigma) and mouse anti-Pan-cytokeratin (Abcam) in room temperature for 3 h or 4°C overnight. Sections were subsequently stained with AF488 rabbit anti-GFP IgG (H+L) antibody (Jackson ImmunoResearch) and AF647 goat anti-mouse IgG (H+L) antibody (Jackson ImmunoResearch) at room temperature for 1 h. All sections were mounted in mounting medium with DAPI (Vector Laboratories). Images were obtained using Leica DMI 6000B wide field microscope.

### Targeted Single-Cell TCR mRNA Sequencing

Cryopreserved splenocytes from the Control group were stained to sort cells into five groups: Tetramer (Tet)^+^ T cells that were CD8^+^, CD4^+^ CD25^+^ CD127^−^, or CD4^+^ CD25^−^ CD127^+^ (*n* = 24 each), and Tet^−^ T cells that were CD8^+^ or CD4^+^ (*n* = 12 each). Cells were sorted directly into RT-PCR buffer (Qiagen) using an Influx cell sorter (Becton Dickinson). Index sorting was performed to collect marker information for each single cell. The sequences of expressed TCRα and TCRβ chains from single cells were obtained by a series of three nested PCR reactions multiple internally nested TCRVα, TCRVβ, TCRCα, and Cβ primers as previously described (19). Reaction products were barcoded and sequencing was performed on a MiSeq System (Illumina). TCR sequencing results were deposited on: https://data.mendeley.com/datasets/99zht5wjtk/1.

### Data Analysis and Statistics

Flow cytometry data were analyzed on FlowJo software (Tree Star). Raw data (FCS files) were deposited on: https://data.mendeley.com/datasets/99zht5wjtk/1.

Data were analyzed using GraphPad Prism 7 and presented as mean values ± SEM. The *p*-value was assessed by two-tailed student's *t*-test or two-way ANOVA, and *p*-values considered significant as follows: ^*^p < 0.05; ^**^p < 0.01, and ^***^p < 0.001. Raw RNA sequencing data were processed and demultiplexed using a custom software pipeline previously described (19).

## Results

### Overexpression of MART1 Peptide Does Not Affect the Overall Chimerism in Humanized Mice

We generated several vectors expressing peptides or MART1 protein containing the peptide sequence ELAGIGILTV from the HLA-A2/MART1 MHC tetramer (Tet). HLA-A2^+^ MART1^−^ melanoma cells were transduced by these different vector variants and tested for their ability to stimulate and be lysed by MART1 TCR-expressing human T cells (Figures S1B–D). Both the protein and one of the peptides (“p1,” thereafter referred to as MART1 peptide) were properly processed and presented to the specific T cells. Lentiviral vectors encoding MART1-TCR and MART1 peptide (Figure 1A) were used to transduce HSCs with high efficiency (Figure S1A) and subsequently two groups of humanized mice were produced as illustrated in Figure 1B. Mice in the Control group received 10^5^ CD34^+^ HSCs transduced with the MART1 TCR and 10^5^ mock-transduced CD34^+^ HSCs; while mice in the Peptide group received 10^5^ CD34^+^ HSCs transduced with the MART1 TCR and 10^5^ CD34^+^ HSCs transduced with the MART1 peptide. The frequency of T cells, B cells, and CD11c^+^ cells, as well as the percentage of MART1-reactive T cells (identified as Tet^+^ cells) were assessed in peripheral blood over time (Figure S1E). Mice in both groups showed ~50% chimerism by 8 weeks after reconstitution (Figure 1C) (a few mice with low chimerism, due to poor grafting of the human thymic tissue, were excluded; Figure S1F). The percentage of human T cells steadily increased in the blood over time as the thymic graft developed (Figure 1D), resulting in a relative decrease in the frequency of human B cells (Figure 1E). The percentage of human CD11c^+^ cells remain stable over time at ~4% of CD45^+^ cells (Figure 1F). Importantly, there was no significant difference between the two groups in the overall frequency of immune cell populations. In the Peptide group, the percentage of GFP^+^ cells (MART1 peptide-expressing cells) hovered around 30–40% in B cells and CD11c^+^ cells (Figure 1G). GFP expression in T cells increased over time as new HSC-derived T cells replaced thymocytes that preexisted in the thymic tissue (Figure S1G). The CD4/CD8 T cell ratio decreased in the Control group (Figure 1H), because of the high frequency of Tet^+^ cells that develop as CD8^+^ T cells. There was no significant difference in the frequency or phenotype of MART1-TCR^+^ T cells between mice with or without thymectomy (indicating that the majority of the Tet^+^ T cells were generated in the human thymic graft in this model), or between NSG and HLA-A2 Tg NSG mice (data no shown). In fact, the human thymic graft contributed ~99.7% of the total cell numbers and number of human CD45^+^ cells, and ~99.95% of the total human CD8^+^ T cells in combined human and mouse thymi in non-thymectomized mice (Figure S2A).

### MART1 Peptide Expression Leads to a Profound Yet Incomplete Deletion of Specific T Cells

**Figure 2:**
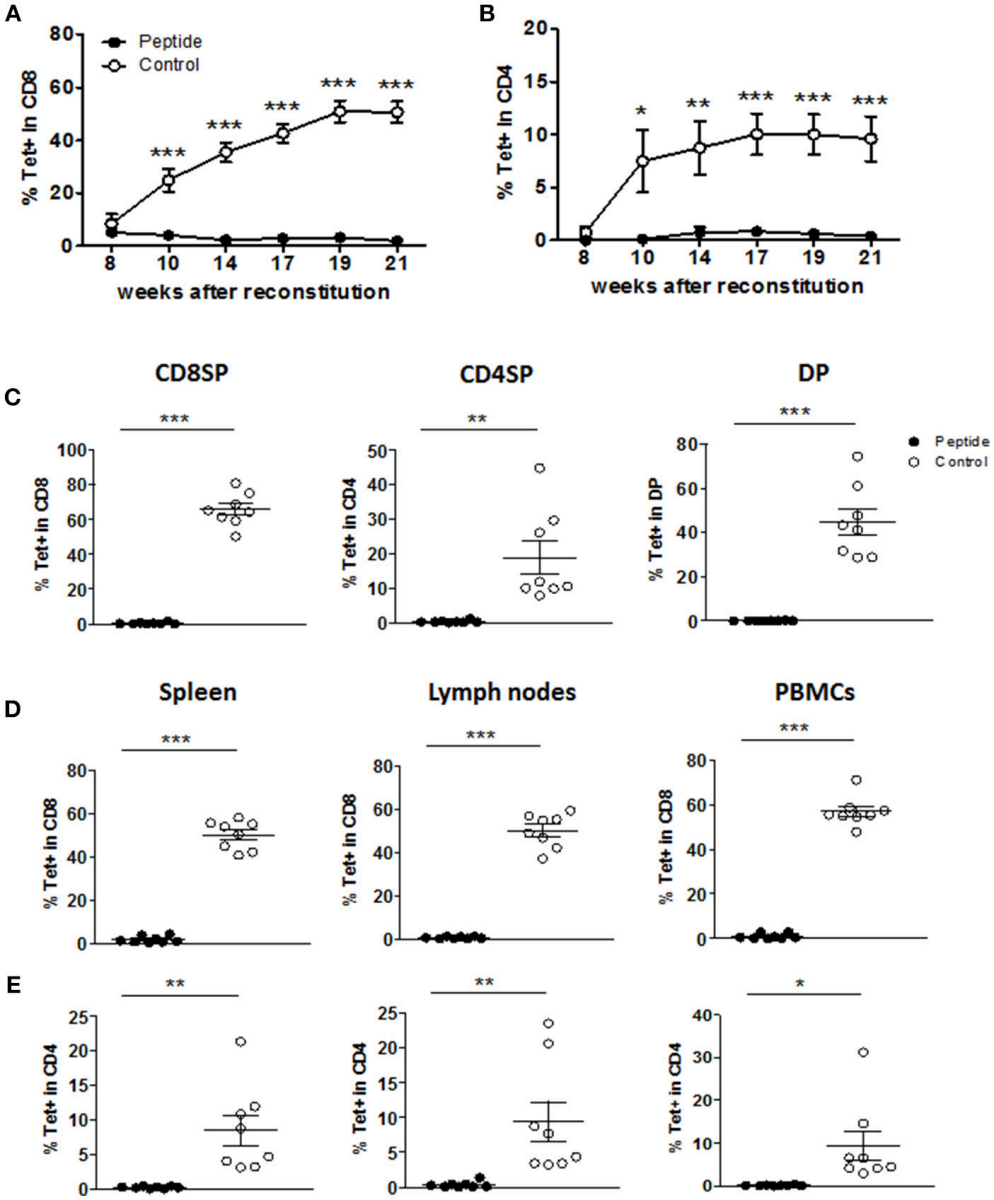
MART1 peptide expression leads to a profound deletion of specific T cells. **(A**,**B)** Percentage of Tet^+^ cells in human CD8^+^ T cells **(A)** and CD4^+^ T cells **(B)** in the peripheral blood at different time points after HSC and thymic graft transplantation (mean ± SEM; peptide *n* = 8, control *n* = 9). **(C**–**E)** Percentage of Tet^+^ cells in different thymocyte populations **(C)**, and in CD8^+^ T cells **(D)** and CD4^+^ T cells **(E)** from peripheral tissues and blood at end point (mean ± SEM, peptide *n* = 8, control *n* = 8). All panels: filled and open circles depict mice from Peptide and Control groups, respectively.

MART1-reactive T cells were tracked by staining with the HLA-A2/MART1 Tet. In the Control group, Tet^+^ CD8^+^ T cells were released into the blood and accumulated, reaching a plateau at nearly 50% of all CD8^+^ T cells (Figure 2A). In addition, some of the Tet^+^ cells developed as CD4^+^ T cells (<10% of total CD4^+^ T cells; Figure 2B). In contrast, the frequency of mature Tet^+^ CD8^+^ or CD4^+^ T cells in the blood was severely reduced in the Peptide group (Figures 2A,B). Starting at 24 weeks post-reconstitution, mice were sacrificed and immune cell populations in the thymic graft, spleen, lymph nodes, and blood were analyzed. The number of cells recovered from the different tissues did not differ between the two groups (Figure S2B). Although humanized mice tend to have underdeveloped lymph nodes with abnormal lymphoid architecture, we were nonetheless able to recover most lymph nodes from reconstituted mice (Figure S2C). The lymph nodes from the Peptide group tended to yield more cells (Figure S2B), perhaps due to the higher CD4/CD8 ratio in the Peptide group (Figure 1H) combined with the higher CD4/CD8 ratio in lymph nodes relative to spleen (Figure S2D) (20). The deletion of MART1-reactive T cells in the Peptide group was evident in all Tet^+^ thymocyte populations (Figure 2C) as well as in spleen, lymph nodes and peripheral blood mononuclear cells (PBMCs), both in CD8^+^ T cells (Figure 2D) and CD4^+^ T cells (Figure 2E). These data suggest that recognition of the MART1 peptide during thymic development led to efficient albeit incomplete purging of autoreactive T cells (from ~65% of CD8^+^ T cells to <0.5% in average in the thymus). As a result, only a few mature Tet^+^ CD4^+^ and CD8^+^ T cells in Peptide group were detected in the periphery.

### HSCs Generate All Major Human APC Populations in Humanized Mice

**Figure 3:**
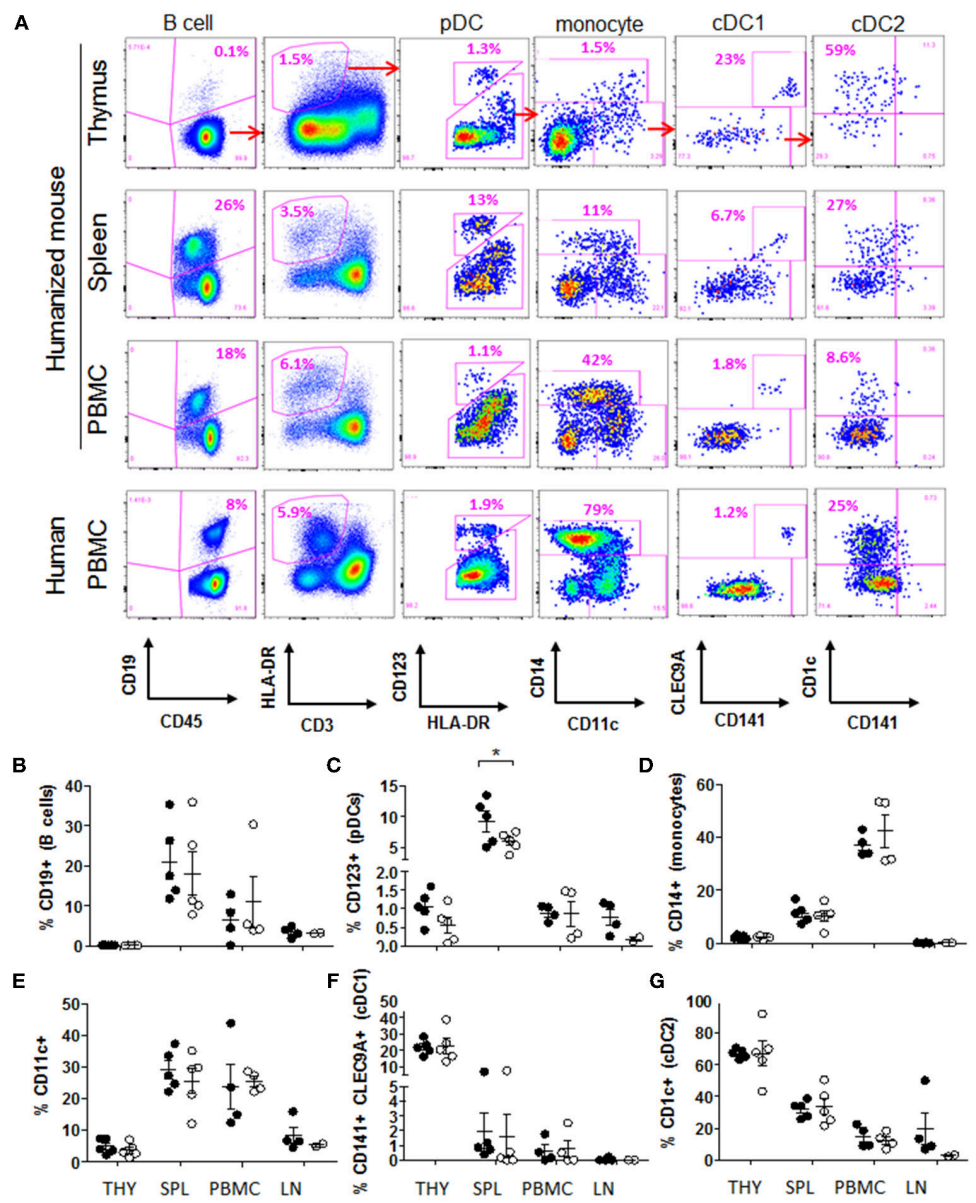
HSCs generate all major human APC populations in humanized mice. **(A)** Flow cytometric identification of major type of APCs in the thymic graft, spleen and peripheral blood of humanized mice at end point (example from Peptide group); and in the blood of a healthy human donor. **(B–G)** Relative frequency of APC populations in Peptide group (closed circles) and Control group (open circles): **(B)** CD19^+^ B cells (gated on CD45^+^ cells), **(C)** CD123^+^ pDCs (gated on CD45^+^ HLA-DR^+^ CD3^−^ cells), **(D)** CD14^+^ monocytes (gated on CD45^+^ HLA-DR^+^ CD123^−^ cells), **(E)** CD11c^+^ cells (gated on CD45^+^ HLA-DR^+^ CD123^−^ cells), **(F)** CD141^+^ CLEC9A^+^ cDC1 cells (gated on CD11c^+^ cells), and **(G)** CD1c^+^ cDC2 cells (gated on CD11c^+^ CLEC9A^−^ cells). All graphs show the mean ± SEM from *n* = 5 mice per group (thymus and spleen); *n* = 4 mice per group (PBMC); *n* = 4 (lymph nodes from Peptide group) and *n* = 2 (lymph nodes from Control group). *t*-test comparison showed no difference between groups, except where indicated. THY, thymus; SPL, spleen; PBMC, peripheral blood mononuclear cells; LN, lymph nodes.

In order to better appreciate the extent of antigen presentation that led to such clonal deletion, and the nature of APCs that might be involved, we performed a flow cytometry analysis of APC subsets in thymic graft, PBMCs, spleen and lymph nodes. We could identify all major human APC populations in these tissues, including B cells, monocytes, plasmacytoid dendritic cells (pDCs), as well as the CD141^+^ CLEC9A^+^ and CD1c^+^ subsets of conventional DCs (referred to as cDC1 and cDC2, respectively) (Figure 3A). The frequency of these populations in the blood of humanized mice was comparable to what is seen in humans (Figure 3A), and there was no significant difference in frequency between the two groups in the various tissues examined, with the exception of spleen pDCs (Figures 3B–G). The tissue distribution of APC subsets was consistent with what is typically seen in humans and immunocompetent mice, with paucity of B cells in the thymus, and with monocytes and pDCs primarily populating the peripheral blood and spleen, respectively (Figures 3B–D). While CD11c^+^ cells were found at a higher frequency in spleen and blood than in the thymus, those in the thymic graft displayed a more fully differentiated cDC1 and cDC2 phenotype, indicating that the human thymic tissue more optimally supported the development and maturation of these DC subsets, making them capable of efficient negative selection of thymocytes (Figures 3E–G). Importantly, 20–40% of APCs in tissues from the Peptide group expressed GFP and therefore the MART1 peptide (Figures S3A–D). In addition, developing thymocytes constituted an additional source of peptide, which could be released into the thymic environment during apoptosis for presentation by local APCs (Figures S1G, S3E–G). Thus, in this model, the antigen is highly abundant within the thymus.

### Levels of TCR, CD3, CD8, and CD4 Are Reduced on Thymocytes Exposed to Antigen

**Figure 4:**
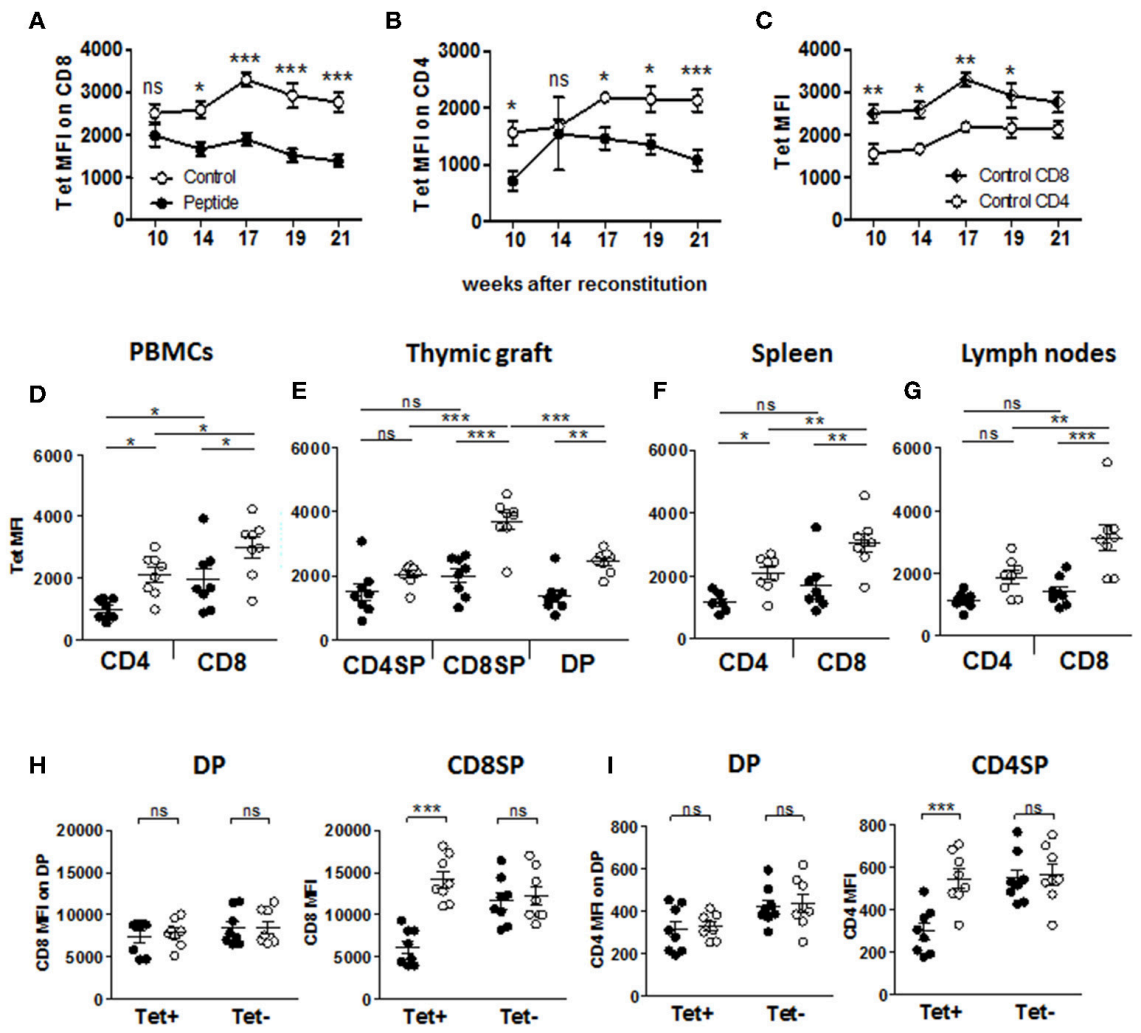
Expression of the TCR and accessory molecules CD4 and CD8. **(A**–**C)** Flow cytometric of MART1-TCR level by mean fluorescence intensity (MFI) of tetramer staining in Tet^+^ CD8^+^ T cells **(A)** and Tet^+^ CD4^+^ T cells **(B)** comparing Control vs. Peptide groups, and in Control group **(C)**, comparing CD8^+^ and CD4^+^ Tet^+^ T cells at different time points after reconstitution (mean ± SEM, peptide *n* = 8, control *n* = 9). **(D**–**G)** Tetramer staining MFI of Tet^+^ T cells from PBMCs **(D)**, thymic graft **(E)**, spleen **(F)**, and (lymph nodes **(G)** at end point (mean ± SEM, peptide *n* = 8, control *n* = 8). **(H)** CD8 expression (MFI) on single and double-positive Tet^+^ and Tet^−^ CD8^+^ T cells in thymic graft. **(I)** CD4 expression (MFI) on single and double-positive Tet^+^ and Tet^−^ CD4^+^ T cells in thymic graft. All panels: filled and open circles depict mice from Peptide and Control groups, respectively.

Flow cytometric analysis of thymocyte populations (Figure S4A) revealed a significant decrease in CD8^+^ single-positive (SP) cells in the Peptide group as compared to the Control group (Figure S4B), in line with the difference in CD4/CD8 ratio seen in the periphery (Figure 1H), while all other thymic populations were unaffected. Furthermore, SP thymocytes expressed significantly higher levels of CD3, CD4, and CD8 (based on mean fluorescence intensity) than the double-positive (DP) thymocytes, and CD4^+^ T cells expressed significantly more CD3 than CD8^+^ T cells in both groups, while the levels of these molecules did not differ between the two groups (Figures S4C–E). However, the few remaining MART1-reactive (i.e., Tet^+^) CD8^+^ and CD4^+^ T cells found in the blood of mice in the Peptide group displayed significantly lower Tet binding (i.e., MART1-TCR level) than their equivalent in the Control group (Figures 4A,B,D). Reduced MART1-TCR levels in the Peptide group were also seen in thymus, spleen and lymph nodes, more consistently in the CD8^+^ T cell population (Figures 4E–G). Tet binding was also consistently lower on CD4^+^ T cells as compared to CD8^+^ T cells in all analyzed tissue compartments (Figures 4C–G), a difference that was most pronounced in the Control group. In the presence of antigen, MART1-reactive thymic CD8SP T cells, but not CD4SP or DP T cells also expressed lower CD3 levels (Figure S4F). The expression of CD8 increased during the transition from DP to CD8SP, unless the T cells had been exposed to antigen (Figure 4H), and the same was true for the expression of CD4 between the DP and CD4SP stages (Figure 4I). However, differences in the level of CD8 and CD4 expression normalized after emigration to the periphery (Figures S4G,H), except in lymph nodes where CD8 expression on antigen-experienced Tet^+^ CD8^+^ T cells remained significantly lower (Figure S4G). Altogether, these data indicate that MART1-reactive T cells either downregulated or failed to upregulate TCR, CD3, CD4, and CD8 in the presence of autoantigen during thymic development.

### Phenotype of MART1-Reactive T Cells in the Presence or Absence of Antigen

**Figure 5:**
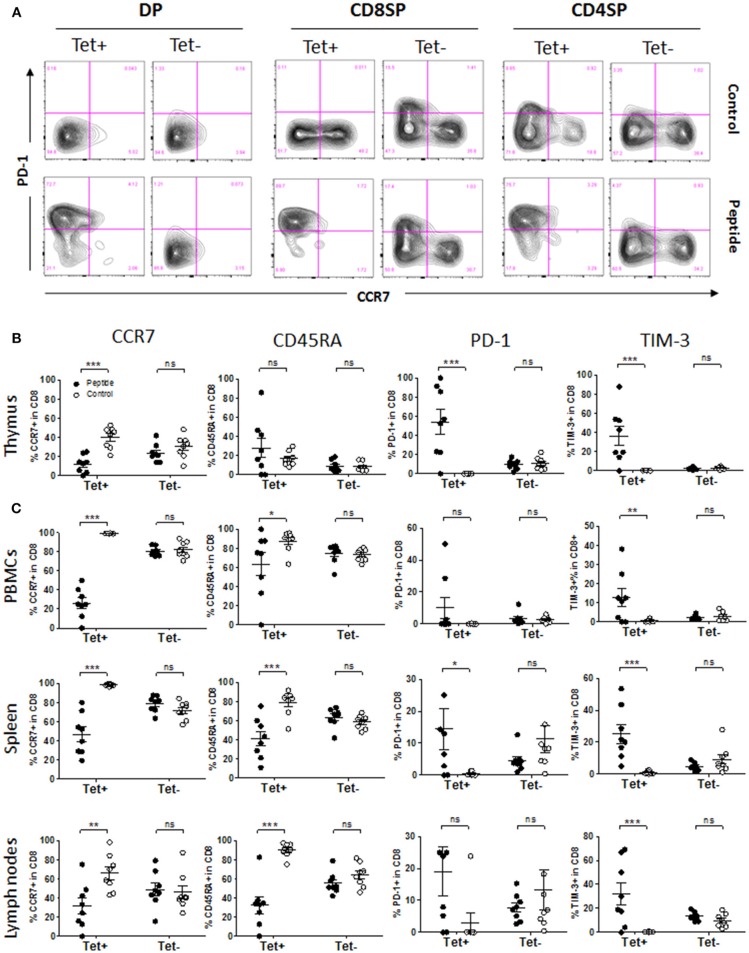
Phenotype of MART1-reactive T cells in the presence or absence of antigen. **(A)** Representative flow cytometry plots of CCR7 vs. PD-1 staining on Tet^+^ and Tet^−^ cells among DP, CD8SP, and CD4SP thymocytes from Control group (upper row) and Peptide group (lower row) at end point. **(B**,**C)** Percentage of CCR7^+^, CD45RA^+^, PD-1^+^ and TIM-3^+^ cells among Tet^+^ CD8^+^ and Tet^−^ CD8^+^ T cells from Control group (open circles) and Peptide group (filled circles) in thymic graft **(B)**, PBMCs, spleen, and lymph nodes **(C)** at end point (mean± SEM, *n* = 8 mice per group).

During thymic development, polyclonal (Tet^−^) human immature T cells upregulated either PD-1 or CCR7 during the transition from DP to SP stage, indicating two separate outcomes possibly determined by whether antigen was encountered or not (Figure 5A). Approximately 10–15% of CD8SP and 5% of CD4SP among Tet^−^ cells expressed PD-1 at a given time, reflecting those that are poised for apoptosis (Figures 5A,B and Figures S5A–C). Expression of CD45RA followed CCR7 upregulation during that transition (Tet^−^ cells; Figures S5A–C). The phenotype of MART1-reactive (Tet^+^) CD8^+^ T cells was clear-cut, becoming either PD-1^−^ CCR7^+^ in the absence of antigen or PD-1^+^ CCR7^−^ in the presence of antigen, and the same was true for Tet^−^ T cells (Figure 5A). Upregulation of CD45RA in Tet^+^ thymocytes took place independently of CCR7 expression and the presence of MART1 peptide (Figures S5B,C). PD-1 upregulation was evident as early as at the DP stage in Tet^+^ T cells, while it was only observed at the SP stage in Tet^−^ T cells (Figure 5A and Figures S5A–C). Upon antigen encounter, some of the MART1-reactive CD8^+^ and CD4^+^ thymocytes upregulated TIM-3 in addition to PD-1 (Figure 5B and Figures S5A–C). In contrast to Tet^+^ CD8^+^ T cells, some Tet^+^ CD4^+^ T cells became PD-1^+^ rather than CCR7^+^ in the absence of MART1 peptide, similar to some Tet^−^ T cells (Figure 5A), indicative of an alternative antigen being recognized. These data demonstrate extensive and contrasting phenotypic changes in human T cells based on autoantigen encounter during thymic development. In the periphery, MART1-reactive CD8^+^ T cells in the Control group maintained a naïve phenotype, with more of them expressing CCR7 and CD45RA compared to the Tet^−^ T cell population, while antigen-exposed Tet^+^ CD8^+^ T cells had a significantly fewer CCR7^+^ and CD45RA^+^ cells, indicating a clear transition from naïve to effector/memory phenotype (Figure 5C and Figures S5D–F). However, there were fewer PD-1^+^ and TIM-3^+^ T cells in the periphery of mice from the Peptide group (Figure 5C) compared to the thymus (Figure 5B). The phenotype of Tet^−^ T cells was unchanged in all examined tissues (Figure 5). These phenotypes were also seen earlier, between 14 and 21 weeks after reconstitution (Figure S5G).

### MART1-Reactive Regulatory T Cells Are Deleted by Hematopoietic APCs

**Figure 6:**
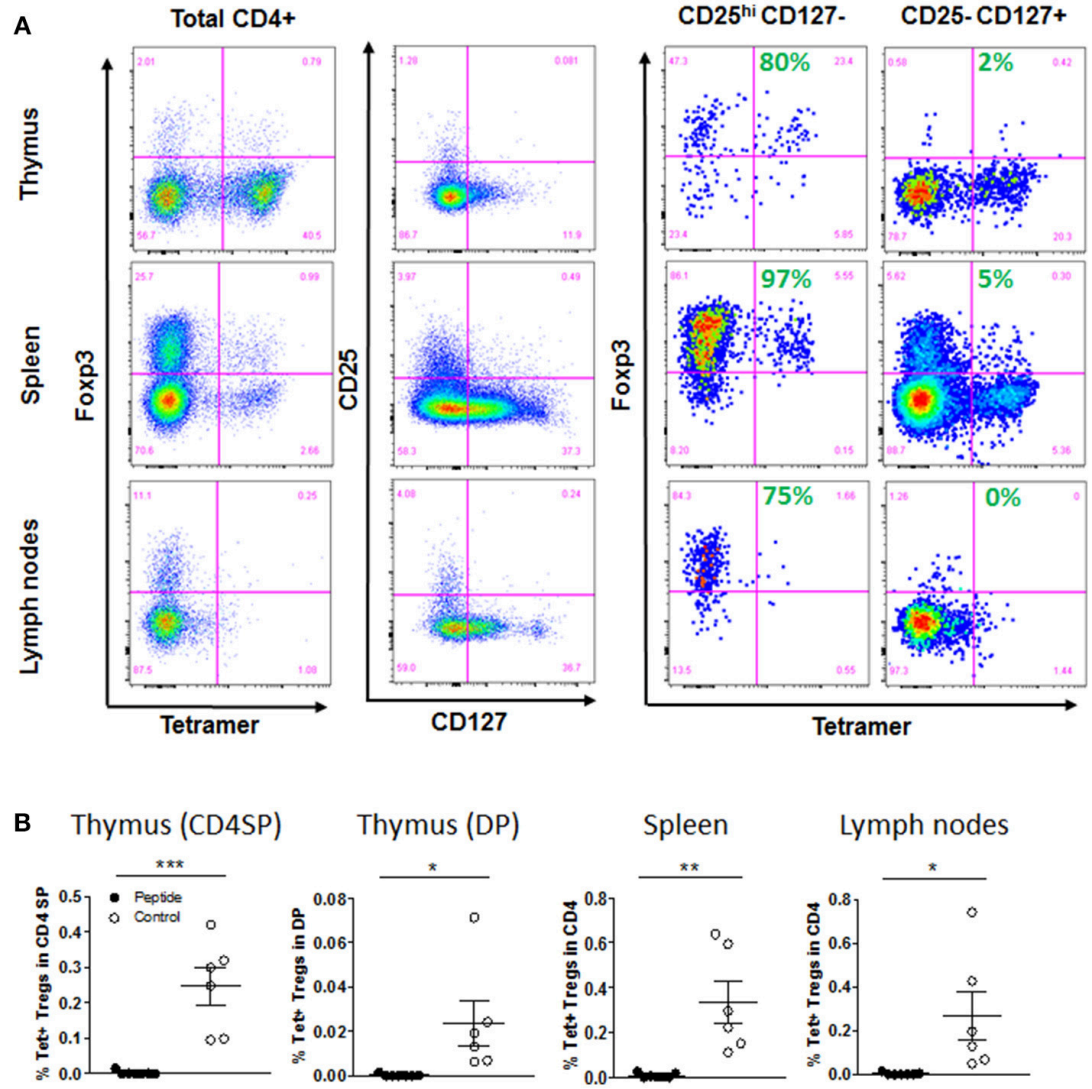
Selection of regulatory T cells. **(A)** Gating strategy for thymic, spleen and lymph node Treg cells populations, after first gating on live, singlet, CD45^+^, CD3^+^, and CD4^+^ cells: Foxp3 vs. tetramer staining after gating on total CD4^+^ T cells, or on CD25^hi^ CD127^−^ or CD25^−^ CD127^+^ among CD4^+^ T cells (Control group shown). Shown in green is the percentage of Foxp3^+^ cells within Tet^+^ T cells. **(B)** Flow cytometry analysis showed percentage of Tet^+^ Tregs (Foxp3^+^ CD25^hi^ CD127^−^) among CD4SP and DP cells in thymic graft, or CD4^+^ T cells in spleen and lymph nodes [mean ± SEM, Control group (open circles) *n* = 6; Peptide group (filled circles) *n* = 7].

Because some MART1-reactive T cells developed as CD4^+^ T cells, we wondered if the recognition of antigen would lead to positive selection of some of these cells as Tregs. Indeed, we identified Foxp3^+^ within Tet^+^ CD4^+^ T cells, and as expected, these were mostly confined to the CD4^+^ CD25^+^ CD127^−^ T cell population (Figure 6A and Figure S6A). However, to our surprise, it is in the absence of antigen that these Tregs could develop (again, indicating recognition of an alternative antigen), while they were completely deleted in the Peptide group (Figure 6B). There was no difference in the overall Treg population in the thymic graft and spleen, but mice in the Peptide group had almost twice as many non-specific Tregs in the lymph nodes (Figure S6B).

### Dual TCR Expression Influences the Development of MART1-Reactive T Cells as CD4^+^

**Figure 7:**
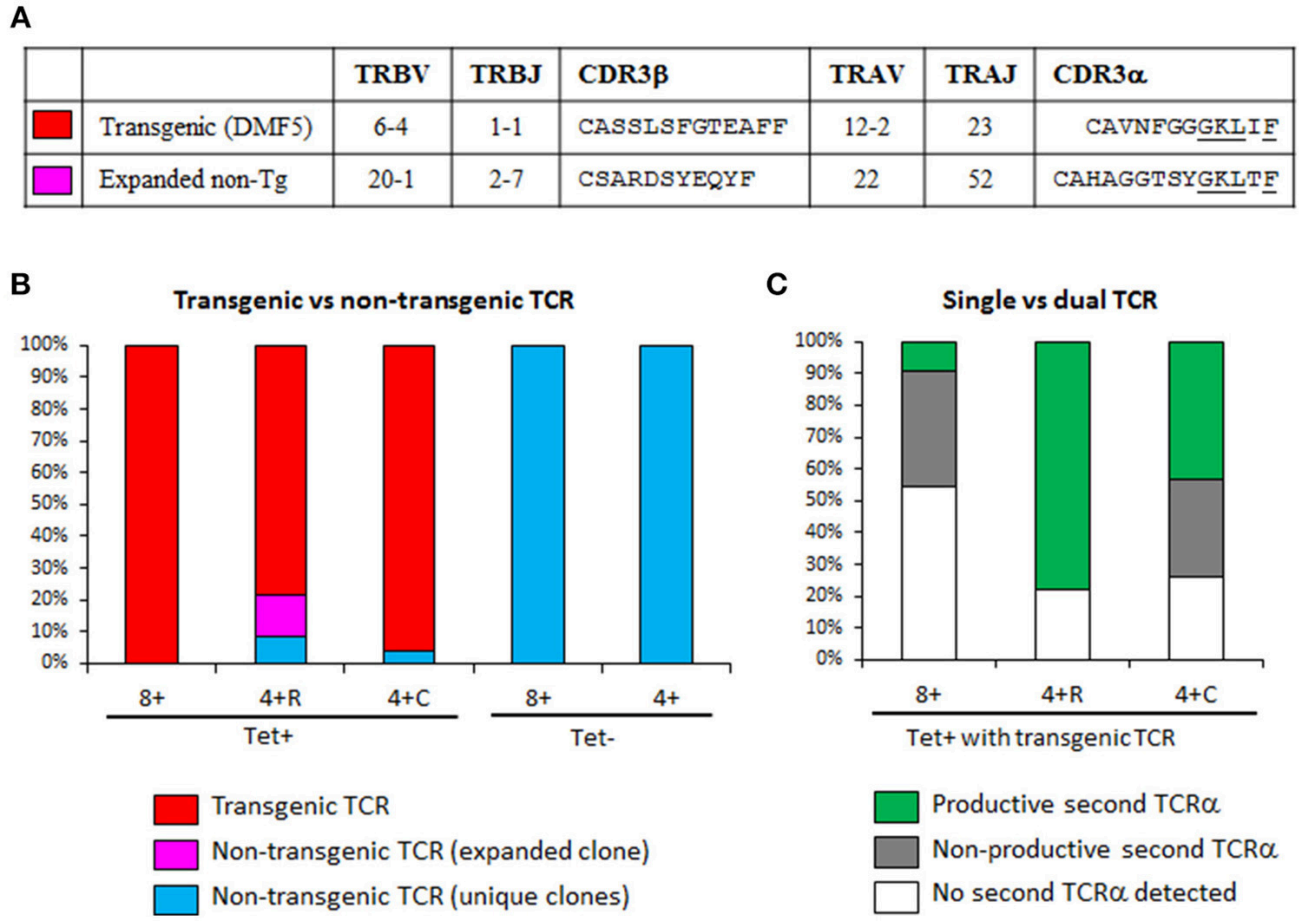
Transgenic T cells expressing a productive second TCR are more likely to become CD4^+^. **(A)** TCRα and TCRβ usage of HLA-A2/MART1-reactive TCRs repeatedly identified by single cell TCR sequencing. The transgenic TCR is the DMF5 clone introduced by lentiviral transduction; while the expanded non-transgenic TCR was from a Tet-binding CD4^+^ T cell clone, identified three times in a sample of 23 cells. CDR3 homology that might contribute to similar binding to MART1 Tet is underlined. **(B)** Percentage of cells expressing the transgenic TCR (clone DMF5) or a non-transgenic TCR in sorted T cell populations. **(C)** Percentage of cells with transgenic TCR that express a productive second TCRα, a non-productive second TCRα, or had no second TCRα detected (the last two equivalent to single TCR). Lack of two productive TCRβ was used to exclude the possibility of having two T cells in the same well. The efficiency of TCR amplification was somehow low in Tet^+^ CD8^+^ T cells (11/24), but high in other subsets: Tet^+^ CD4^+^ CD25^+^ CD127^−^ (23/23), Tet^+^ CD4^+^ CD25^−^ CD127^+^ (24/24), Tet-CD8^+^ (10/12), and Tet^−^ CD4^+^ (12/12). The few Tet^+^ cells that were not TCR-transgenic were not included in this analysis as to not confound the results. 4 + R, CD4^+^ CD25^+^ CD27^−^ (regulatory); 4 + C, CD4^+^ CD25^−^ CD127^+^ (conventional).

Our above observations that some Tet^+^ CD4^+^ T cells underwent negative selection (PD-1 induction) or positive selection as Tregs in the absence of MART1 peptide led us to hypothesize that these T cells may be more likely to have a second TCR on their surface that is MHC class II restricted (skewing development to CD4^+^ T cells) and directed to another self-antigen (resulting in central deletion or Treg selection). Thus, we performed single-cell TCR mRNA sequencing of cryopreserved splenocytes from the Control group (19). We did a sampling of three populations of MART1-reactive T cells (Tet^+^ CD8^+^, Tet^+^ CD4^+^ CD25^+^ CD127^−^, and Tet^+^ CD4^+^ CD25^−^ CD127^+^) as well as some Tet^−^ CD8^+^ and CD4^+^ T cells as control (Figure S7A). Previous staining of the same sample indicated that the CD25^+^ CD127^−^ and CD25^−^ CD127^+^ fractions contained 97 and 5% Foxp3^+^ cells respectively (Figure 6A). Index sorting data confirmed the expected expression of CD8, CD4, Tet, CD25, and CD127 on the sorted subsets (Figure S7B). We found that 100% of Tet^+^ CD8^+^ cells with amplified TCR had the Tg-TCR (clone DMF5; Figures 7A,B), but not all Tet^+^ CD4^+^ cells did (4–22% expressed a different TCR), indicating that these cells may be naturally MART1-reactive or cross-reactive (Figure 7B). Interestingly, one such clone was found multiple times in the CD25^+^ CD127^−^ population, contributing 13% to this population (Figures 7A,B). Importantly, none of the Tet^−^ cells expressed the Tg-TCR (Figure 7B). Moreover, we detected a second productive TCRα in up to 25% of control (Tet^−^) T cells (Figure S7C), consistent with what was previous previously reported in human and mouse T cells (21–23). However, a striking difference was observed within MART1-reactive T cells expressing the Tg-TCR, whereby a second productive TCRα was detected in only few CD8^+^ T cells (9%), in substantially more conventional CD4^+^ CD25^−^ CD127^+^ T cells (43%) and in the majority of regulatory CD4^+^ CD25^+^ CD127^−^ T cells (78%) (Figure 7C).

### *Ex vivo* Responsiveness of MART1-Reactive CD4^+^ and CD8^+^ T Cells From Humanized Mice

Both Tet^+^ CD3^+^ CD8^+^ and Tet^+^ CD3^+^ CD8^−^ (CD4^+^) from the spleen were responsive to restimulation with MART1 peptide (sequence of the peptide expressed in Peptide group and used in MHC tetramers) *ex vivo*, with CD4^+^ T cells being less sensitive by ~1 log (Figures S8A,B), possibly due to lower TCR levels. The T cells could respond in absence of exogenous IL-2 if they were naïve (from Control group), but those that were antigen-experienced (from Peptide group) did not respond and died by the end of the 6-day culture (Figures S8C,D). However, in the presence of IL-2, the antigen-experienced T cells could be rescued and responded well to peptide, suggesting that these T cells were indeed anergic (Figures S8C,D). Finally, we find that those T cells were weakly cross-reactive with the native human MART1 peptide (distinct by one amino acid) and with the native mouse MART1 peptide (distinct by two amino acids) (Figure S8E).

## Discussion

The humanized mouse model described here is reminiscent of the many TCR-Tg mice produced and studied in the past, although it is more akin to TCR-retrogenic mice in the way they are produced (i.e., by hematopoietic cell lineage reconstitution in irradiated recipients). The grafted human thymic tissue ensures that T cells and APCs develop and function in an adequate supportive environment. Such model integrating both TCR and antigen constitutes a novel tool to elucidate the development of human autoreactive T cells and to determine to what extent it is comparable to that of murine T cells, which have contributed the basis of our current knowledge on the subject. More specifically, the model presented here is that of an abundant antigen (expressed in a large fraction of hematopoietic cells) recognized with high affinity (the epitope is an A27L mutant of the native MART1_26−35_ epitope found on the HLA-A2/MART1 Tet and to which the DMF5 TCR clone is strongly reactive). The outcome of this engagement is a profound clonal deletion (frequency reduced to <5% of that seen in absence of antigen), including a lack of positive selection of Tregs. This outcome is consistent with recent studies in mice assessing the mechanisms of tolerance in relation to antigen biodistribution (24, 25), which showed that ubiquitous antigens primarily mediate deletional tolerance rather than Treg selection. The lack of complete elimination of autoreactive Tet^+^ T cells that we observed was also consistent with those mouse models. However, it occurred despite the abundance of antigen and the high affinity of the TCR for this antigen.

Our model offers much versatility and potential utility to study factors that may influence the thymic selection and subsequent fate of human autoreactive T cells. Although our high transduction efficiency (Figure S1A) leads to high (beyond physiological) frequency of antigen-specific T cells and of antigen-expressing cells, these can be reduced to very low frequencies by simply diluting transduced HSCs with untransduced HSCs. The strength of the TCR engagement may also be reduced by using a less stimulatory peptide (for example, the responsiveness of our MART1-reactive T cells is about 1,000-fold lower to the native MART1_26−35_ peptide than to the A27L mutant; Figure S8E). Thus, while the present model only allows us to draw conclusions based on this level of antigen abundance and affinity, it can be tweaked in a variety of ways to address physiologically relevant questions. The biodistribution of the antigen may be changed in certain ways. The antigen may be kept strictly peripheral by expressing it in specific mouse tissues and using HLA-Tg NSG mice to make presentation to human T cells possible. Forced antigen expression may not be required if sufficient sequence homology exists between the recognized human peptide and its murine ortholog. The antigen may also be introduced into human HSCs, but using cell-specific promoters driving antigen expression rather than using ubiquitous promoters. As discussed below, this can allow studies aimed at understanding the unique contribution of different human APCs to the response by human T cells in the context of tolerance or immunity, and in an *in vivo* environment. Finally, our study described the thymic development of a T cell clone that normally develops as CD8^+^ T cell. While the TCR is MHC-I restricted, a substantial fraction of the T cells bearing this TCR developed as CD4^+^ T cells, including Tregs. While the relevance of this CD4^+^ T cell population will be discussed below, the use of an MHC-II restricted TCR will be better suited to explore the conditions that impact the fate of human CD4^+^ T cells during thymic development (deletion vs. Treg selection). It will be particularly informative to assess T cell clones that have been identified in autoimmune diseases in relation to whether or not their antigen is expressed in the thymus. As previously mentioned, MART1_26−35_-reactive T cells normally escape thymic deletion despite evidence of MART1 gene expression in human mTECs (3, 4, 7). Conversely, other autoreactive T cells whose antigen is not expressed or presented in the thymus might still be deleted (and/or positively selected as Tregs) (26, 27) by another antigen by cross-reactivity or via a second TCR (discussed below). Thus, studies performed so far to assess autoantigen expression in mTECs from human thymic tissue provide only an incomplete understanding of the process of autoreactive T cell selection and cannot reliably predict the extent to which a specific autoreactive T cell clone will undergo or escape thymic deletion. This model can more reliably address the fate of specific autoreactive T cells in a normal human thymus. Studies in transplantation tolerance suggest that certain observations made in mice cannot be extrapolated to humans (28). Furthermore, only the use of a human thymic graft can provide the human-specific factors needed for the reliable analysis of human thymocyte development (29).

An important and novel aspect of this model is the introduction of an autoantigen that is expressed in hematopoietic cells in the form of a specific peptide. Because HSCs can give rise to a large panoply of professional APCs, it was essential to document that this model reflects the variety and frequency of APC subsets found in humans tissues (30–32). Several observations support the relevance of this model. First, all major human APC subsets were identified in the different tissues examined from humanized mice. As previously reported in various human tissues (31), cDC2 cells were more frequent than cDC1 cells in all examined tissues. Second, the frequency of HSC-derived CD11c^+^ cells was stable throughout the life of the mouse. Third, MART1-reactive T cells had no impact on the overall frequency of APC subsets whether they were deleted or not. However, many mice had very few conventional DCs outside the thymus, particularly resident cDC1 cells. This may indicate that the human thymic graft remains an ideal environment for the *in situ* development of human DCs from HSC progenitors and their persistence, due to greater exposure to human growth factors produced by the graft. cDC1 cells in the periphery were affected the most, probably because they develop within the tissue of ultimate residence while migratory cDC2 cells may have developed in a more supportive environment before homing to these sites.

Interaction of autoreactive T cells with autoantigen-expressing/presenting cells, both in the thymus and in the periphery, leads to phenotypic (and functional) changes that may vary based on the type of APC involved, the amount of presented antigen (avidity) and the tissue environment of the interaction. Although the current model does not allow us to delineate subtle differences between APCs given the large number and variety of APCs involved, the presence of all DC subsets in the human thymic graft makes the model capable of recapitulating the nature of thymic selection of developing human autoreactive T cells. With this model, we are able to track a single clone within a complex population of polyclonal T cells comprising cells that undergo or avoid negative selection, and to accurately relate phenotypic changes to actual antigen encounter events (comparing two “extremes” marked by the complete absence vs. abundant presence of autoantigen). For example, the data on polyclonal (Tet^−^) cells show that T cells upregulate either CCR7 or PD-1 when transitioning from DP to SP stage (Figure 5A), but Tet^+^ T cells help us to firmly establish that PD-1^+^ cells are those that encountered antigen, while CCR7^+^ cells are those that did not. In Tet^+^ T cells, PD-1 upregulation was early (starting at DP stage) and robust, while TIM-3 was up-regulated to a more limited extent. These data are consistent with the report that PD-1 upregulation occurs at the CCR7^−^ stage in mice (33). In absence of antigen, thymocytes progressed from CCR7^−^ CD45RA^−^ to CCR7^+^ CD45RA^−^ and then to CCR7^+^ CD45RA^+^ prior to exiting the thymus. CCR7 is upregulated during positive selection and is required for migration to the medulla in mice (34, 35). Interestingly, we did not observe CCR7^+^ PD-1^+^ T cells in both Tet^+^ and Tet^−^ populations, suggesting that either PD-1 was induced prior to migration to the medulla and prevented CCR7 induction or that thymocytes rapidly downregulated CCR7 after homing to the medulla and encountering antigen. Alternatively, human thymocytes may use other means to migrate to the medulla, such as the CCR9-CCL25 axis (36). In the spleen and blood, these cells remained CCR7^+^ CD45RA^+^, but in the lymph nodes, many down-regulated CCR7 (this was unexpected for a population of naïve T cells, and whether this is biologically relevant or an artifact of humanized mouse will need to be addressed). In mice from the Peptide group, the expression of PD-1 was less pronounced in the peripheral tissues than in the thymus, while that of TIM-3 was more evident in the periphery. Expression of PD-1 and TIM-3 on peripheral T cells is associated with tolerance, anergy and/or exhaustion. We found that T cells from mice in Peptide group were indeed anergic (hyporesponsive to *ex vivo* peptide restimulation, overcome with exogenous IL-2). These T cells adopted a more heterogeneous phenotype with a mixture of PD-1 and TIM-3 single and double-positive cells. This should not be surprising given the variety of human APCs potentially engaging these T cells, and restricting antigen expression to specific subsets may help us to associate certain T cell phenotypes with the type of APCs they encountered. Despite the extensive presence of antigen, the fraction of MART1-reactive thymocytes that upregulated PD-1 or TIM-3 was not 100% in most mice. Moreover, while these T cells evidently encountered antigen and could undergo deletion already at the DP stage, activated T cells were seen in the SP stage and in the periphery. It is possible that this incomplete deletion is a reflection of the abnormally high frequency of MART1-reactive T cells. This can be easily tested by serially diluting TCR-transduced HSCs with untransduced HSCs. This would clarify whether incomplete deletion of high affinity T cells still occurs under conditions approaching physiological frequency. Understanding the mechanism by which human thymocytes escape thymic deletion is crucial given the presence in “healthy” individuals of circulating autoreactive T cells, many of which are specific to antigens expressed in the thymus (37–40).

In our study, we observed that the few remaining Tet^+^ T cells in the Peptide group had reduced expression of TCR (in CD8^+^ T cells, and in some tissues, in CD4^+^ T cells), of CD3 (in CD8^+^ T cells), of CD4 (in CD4^+^ T cells) and of CD8 (in CD8^+^ T cells). Because these markers were found to be increased between the DP and SP stages in the Control group, we propose three possible scenarios: (1) antigen encounter prevents the upregulation of these markers, (2) antigen encounter induces the downregulation of these markers (33), and/or, (3) these few cells constitute a subset of MART1-reactive T cells that circumvented thymic deletion by a selective advantage conferred by these markers being collectively expressed at low (insufficient) levels prior to antigen encounter. Indeed, low levels of TCR and CD3 can minimize the stimulation of thymocytes that would otherwise result in apoptosis. Furthermore, negative selection is also dependent of the coreceptors CD4 and CD8, in part by enabling the recruitment of Lck (41, 42). We also observed that although CD3 levels increase from DP to SP stages, they were always significantly higher in CD4^+^ than in CD8^+^ T cells, both in SP thymocytes and in the periphery. Others have also reported higher CD3 levels in CD4^+^ T cells than in CD8^+^ T cells in human blood (43–45). The lower TCR expression (Tet staining) on the residual T cells may also reflect a MART1-reactive TCR that is non-Tg and with lower affinity.

The Tg-TCR is well-expressed under the MSCV promoter and, as routinely seen in TCR-Tg or retrogenic animals, the introduced TCR is expressed earlier than the endogenous TCR, and this leads to phenotypic changes in the DP stage that are otherwise not seen until the SP stage for regular T cells (e.g., upregulation of PD-1 in presence of autoantigen, or of CD45RA regardless of autoantigen). This is both a caveat and an advantage: a caveat because it does not exactly recapitulate the physiological process of thymic development, and an advantage because it ensures that expression of a second TCRβ is avoided by allelic exclusion. Indeed, expression of two TCRβ is uncommon in normal T cells (46), and even less likely in Tg T cells. Moreover, studies done in humanized mice to produce MART1-reactive T cells have confirmed the absence of non-Tg TCRβ in Tet^+^ T cells (13, 15). However, these studies only looked at the TCRβ and downplayed the fact that TCRα could lead to alternative specificities if sufficiently expressed. By single-cell mRNA-sequencing, we could rule out the presence of two TCRβ (and that of two cells in the well). However, while a small proportion of non-Tg and CD8^+^ TCR-Tg T cells expressed a productive second TCRα chain, within the previously described range (21–23), we observed that CD4^+^ TCR-Tg T cells expressed substantially more, particularly Tregs (nearly 80%), which is consistent with a previous report (47). The fact that not all Tet^+^ CD4^+^ T cells have a productive second TCR may have several explanations, including the possibility that the second TCR was not amplified (limitations of single-cell analysis) and that some CD4^+^ T cells sorted had non-specifically bound the tetramer.

While the production of human antigen-specific T cells through a human thymic graft in humanized mice is safer than transducing mature peripheral CD8^+^ T cells because of efficient allelic exclusion of the endogenous TCRβ (13, 15), we show that this approach is not completely devoid of potential off-target effects due to a second TCR found also in a fraction of these T cells. Interestingly, the presence of the endogenous TCRα seems to be a factor supporting the development of Tet^+^ T cells as CD4^+^ T cells despite the expression of an MHC-I restricted TCR, and having two competing TCRs may also explain why the level of Tet staining is lower in Tet^+^ CD4^+^ T cells than in Tet^+^ CD8^+^ T cells. While we do not know the MHC restriction of the second TCR, it is tempting to speculate that the few TCR-Tg CD8^+^ T cells that have two TCRα may have both of them MHC-I restricted, while TCR-Tg CD4^+^ T cells that have two productive TCRα may be MHC-I restricted through the Tg-TCRαβ and MHC-II restricted through the second TCR formed by Tg-TCRβ and endogenous TCRα. However, the second TCR may not entirely explain the skewed development toward CD4^+^ T cells, as there was still a notable fraction of Tet^+^ CD4^+^ for which a second TCRα was not amplified, suggesting that either the approach has limited sensitivity or a second TCRα is not absolutely required for TCR-Tg Tet^+^ T cells to become CD4^+^. Whether one T cells can have both MHC-I and MHC-II restriction via two TCRs is an interesting concept that warrants further exploration. On the one hand, a T cell clone has previously been reported to be restricted to both MHC-I and MHC-II in mice (48), but this was ascribed to cross-reactivity because the dual restriction was maintained on a RAG2-deficient background (which eliminates endogenous TCRs). Our expanded non-Tg CD4^+^ T cell clone that repeatedly bound the MHC-I Tet (thereby ruling out non-specific binding) had the same non-productive second TCR (thus no dual TCR), suggesting that MHC-I/MHC-II cross-reactive T cells can also naturally develop in humans. On the other hand, in the case of MHC-I/MHC-II-reactive T cells with dual TCR, it is still possible that the MHC-II restricted TCR drives the positive selection of the developing T cells as CD4^+^ when MHC-II/CD4 signals are stronger overall than MHC-I/CD8 signals. Finally, expression of a second TCR can affect Tregs in different ways. First, it doubles the chance of autoantigen encounter and positive selection as Tregs. More importantly, when a self-reactive TCR is “diluted” by another TCR on the cell surface, this diminishes the avidity of interaction during self-antigen encounter. Consequently, a self-reactive T cell that would otherwise be deleted may be rescued by becoming a Treg through more limited TCR signaling. When the TCR does not see a self-antigen and is not engaged in the thymus, having a second TCR would not affect thymic development. Altogether, this may explain why nearly 80% of MART1-reactive Tregs in the Control group had two TCRs, nearly twice as much as their non-Treg counterparts (47). On the flip side, this reduced avidity may also prevent T cells with lower TCR affinity (that would otherwise be positively selected as Tregs) from receiving sufficient stimulation to become Tregs, with possible implications on the risk of autoimmunity (23).

The overall frequency of Tet^+^ CD8^+^ T cells appears to be another factor influencing the emergence of Tet^+^ CD4^+^ T cells, as their relative frequency are correlated, when combining data from our study and two others using this MART1-reactive TCR (13, 14) (Figure S7D; Table S1). Moreover, in studies featuring high frequency of Tet^+^ CD8^+^ T cells (40–80% of CD8^+^), the Tet staining on CD8^+^ T cells was quite high in fluorescence intensity (well-separated) relative to Tet^−^ T cells (Figures S5B,D–F) (13). In contrast, the study with lower frequency of Tet^+^ CD8^+^ T cells (6–37% of CD8^+^) had lower intensity of Tet staining on CD8^+^ T cells and very few CD4^+^ T cells (14). Thus, it is also possible that the development of CD4^+^ T cells is an artifact of the abnormally high frequency of Tg CD8^+^ T cells or that it is linked to the level of transgene expression. This can be tested, again by serially diluting the TCR-transduced HSCs with untransduced HSCs.

The humanized mouse model described here constitutes a powerful tool to study the thymic development of human antigen-specific T cells side-by-side with the general polyclonal thymocyte population, allowing a more accurate dissection of T cell phenotypes in the presence or absence of a specific self-antigens. It may be leveraged to address the extent to which specific autoreactive T cell clones identified in human autoimmune diseases undergo central tolerance, and absent complete thymic deletion, to produce new models of autoimmune diseases featuring one or multiple autoreactive T cell clones and/or to test tolerance-inducing therapies on human T cells *in vivo*.

## Author Contributions

YL, NT, and ST performed experiments and analyzed data. Y-GY and RC designed and directed the research. SR and AH supported single-cell RNA-sequencing studies. YL and RC wrote the manuscript. All authors edited and approved the manuscript.

### Conflict of Interest Statement

The authors declare that the research was conducted in the absence of any commercial or financial relationships that could be construed as a potential conflict of interest.
