# Supplementary material for: Humanized Mice Reveal New Insights Into the Thymic Selection of Human Autoreactive CD8+ T Cells
Source: Front Immunol. 2019 Feb 4;10:63. doi: 10.3389/fimmu.2019.00063 (PMC6369192; doi:10.3389/fimmu.2019.00063)
Supplement: Supplementary file 1 [file Data_Sheet_1.pdf]

Supplementary Material

Humanized Mice Reveal New Insights into the Thymic Selection of Human Autoreactive CD8+ T Cells

Yang Li, Nato Teteloshvili, Shulian Tan, Samhita Rao, Arnold Han, Yong-Guang Yang* and Rémi J. Creusot*

*** Correspondence:** R.J.C. (rjc2150@columbia.edu) and/or Y.G.Y (yy2324@columbia.edu)

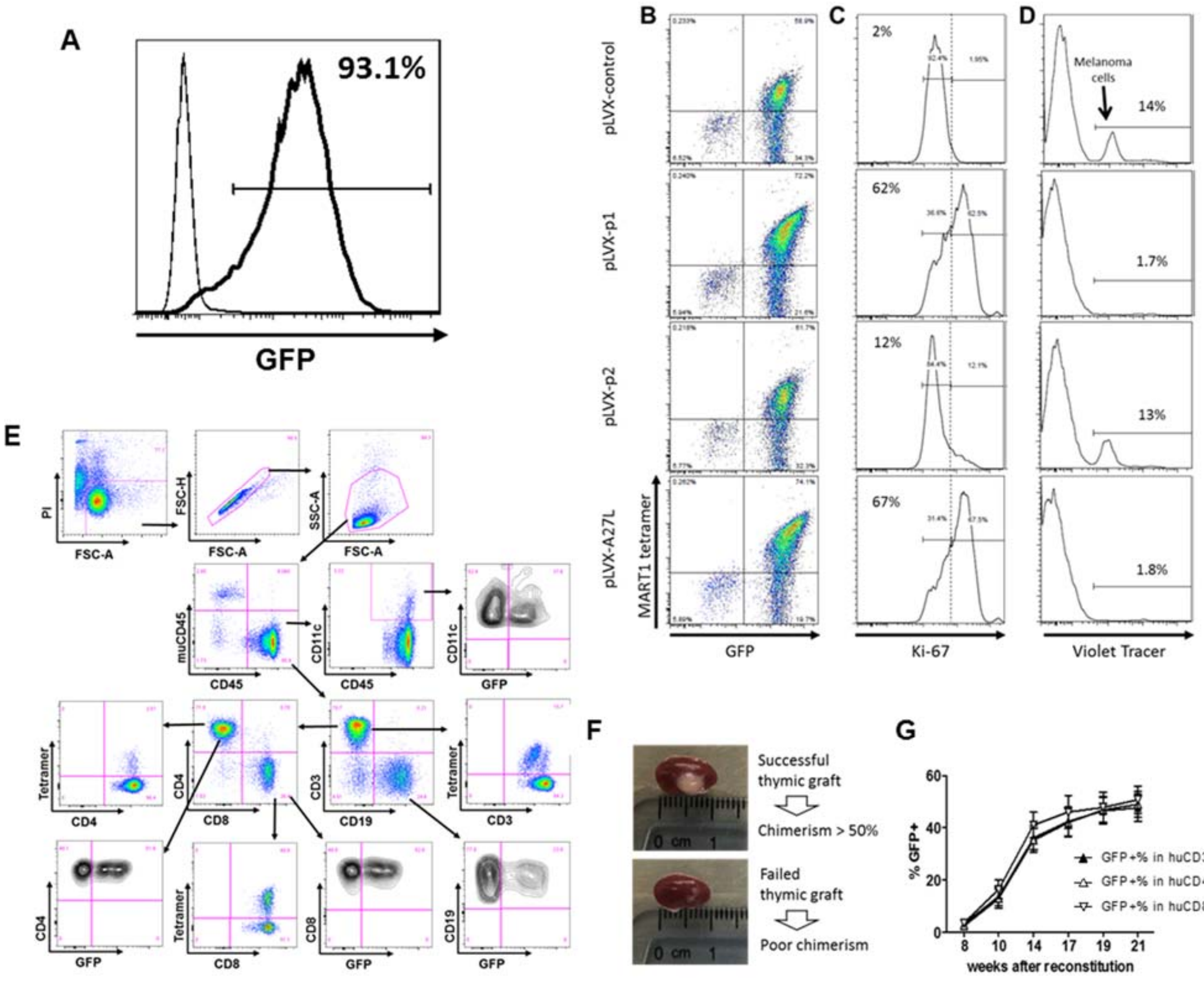

Figure S1: (**A**) Lentiviral vectors transduction efficiency in human HSCs (thin line: MART1-TCR vector, GFP^-; thick line: MART peptide vector, GFP^+). (**B-D**) Validation of MART1 antigen-expressing vectors. HLA-$A2^+$ $MART1^-$ Mel-A375 melanoma cells were transduced with a lentiviral vector (pLVX) expressing antigen and mCherry and labelled with Violet Tracer. Four vectors were used for antigen: control (empty), p1 (MKELAGIGILTVK), p2 (MWKELAGIGILTVFAG) and the MART1 A27L mutant protein. Human peripheral blood mononuclear cells transduced to express MART1 TCR and GFP ($1.2x10^4$ $CD8^+$ T cells) were co-cultured with MART1 antigen-transduced and Violet Tracer-labeled melanoma cells ($1x10^4$). After two days, the cells were analyzed for GFP and MART1 TCR expression (gated on $CD8^+$ T cells, panel (**B**); Ki-67 (gated on Tet^+ GFP^+ T cells, panel (**C**); and the subsistence of melanoma cells, identified as large Violet Tracer positive and CD8- cells panel (**D**). All melanoma cells were still positive for the Violet Tracer after 2 days, as evidenced by the absence of Violet Tracer negative cells with large $CD8^-$ cells. HLA-$A2^+$ $MART1^+$ Mel-624 melanoma cells also stimulated (and were lysed by) MART1 TCR^+ T cells (not shown). (**E-G**): Immune reconstitution of humanized mice. (**E**) Representative gating strategy for the different immune populations (density plots; from Control group), as well as GFP expression (contour plots, from Peptide group). (**F**) Examples of kidneys with grafted thymic tissue in mice that have developed good and poor chimerism. (**G**) % of GFP^+ (MART1 peptide-expressing cells) among $CD3^+$, $CD4^+$ and $CD8^+$ cells measured in the Peptide group (mean ± SEM, n=8).

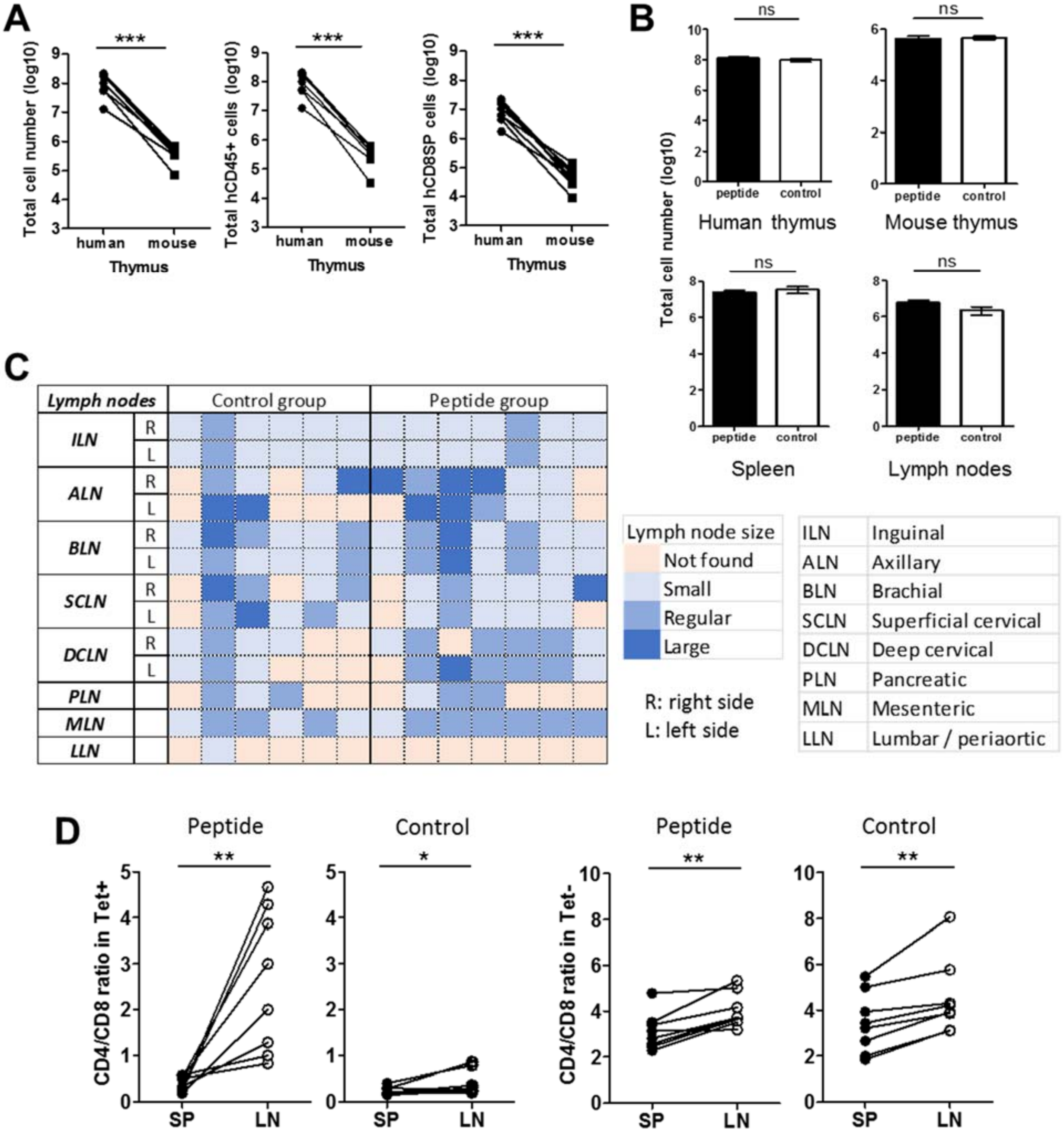

Figure S2: (**A**) Cellularity of human thymic graft versus mouse thymus (non-thymectomized mice, combined data from 4 mice from Control group and 5 mice from Peptide group). Shown are the total number of cells isolated (left), absolute number of human $CD45^+$ cells (middle) and absolute number of human CD8SP (right). Statistical analysis by paired T-test. (**B**) Total number of cells recovered from human thymic graft, mouse thymus, spleen and pooled lymph nodes from humanized mice at end point (mean ± SEM from n= 8 mice per group, except mouse thymus from 4 mice from Control group and 5 mice from Peptide group; unpaired T-test); (**C**) Overview of the different lymph nodes recovered from humanized mice at end point, with the relative size estimated by two investigators in comparison to lymph nodes from 'normal' (NOD) mice. (**D**) Comparison of CD4/CD8 ratio between spleen (SP) and lymph nodes (LN) on Tet^+ cells (left panels) and Tet^- cells (right panels). Statistical analysis by Wilcoxon matched paired T-test.

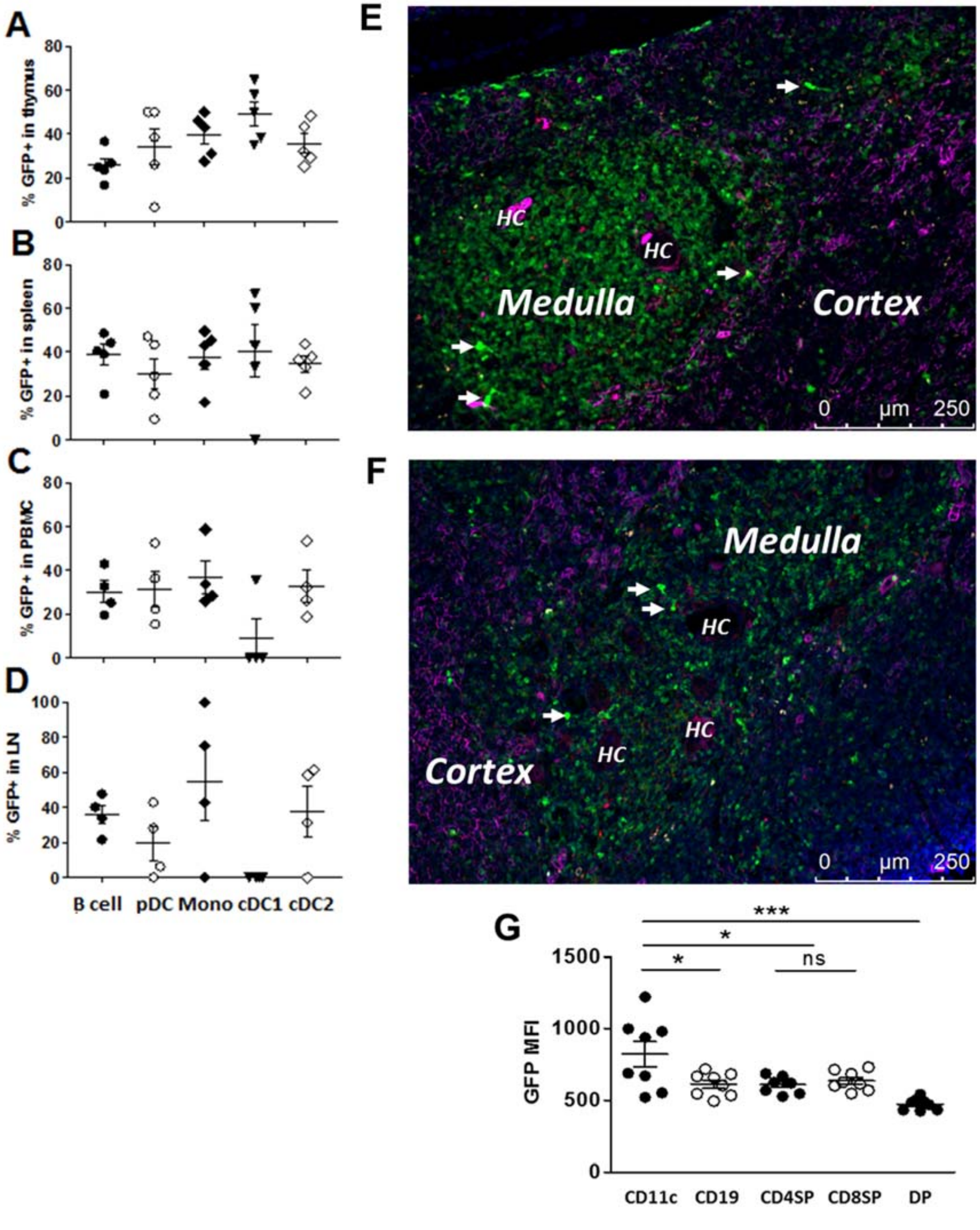

Figure S3: Expression of GFP (reporter for MART1 peptide expression) in different APC populations. (**A-D**) Frequency of GFP$^+$ cells in APC subsets identified as in Fig.3 in thymic graft (**A**), spleen (**B**), PBMCs (**C**) and lymph nodes (**D**) from Peptide group. All graphs show the mean ± SEM from n=4-5 mice. Mono = monocytes. (**E-F**) Immunofluorescence staining of thymic graft sections from Peptide group mice (green GFP, purple pan-cytokeratin). HC indicates Hassall's corpuscles, while arrows point to possible DCs based on brightness (see panel **G**) and morphology. (**G**) Level of GFP expression (GFP MFI) in different immune populations in the thymic graft.

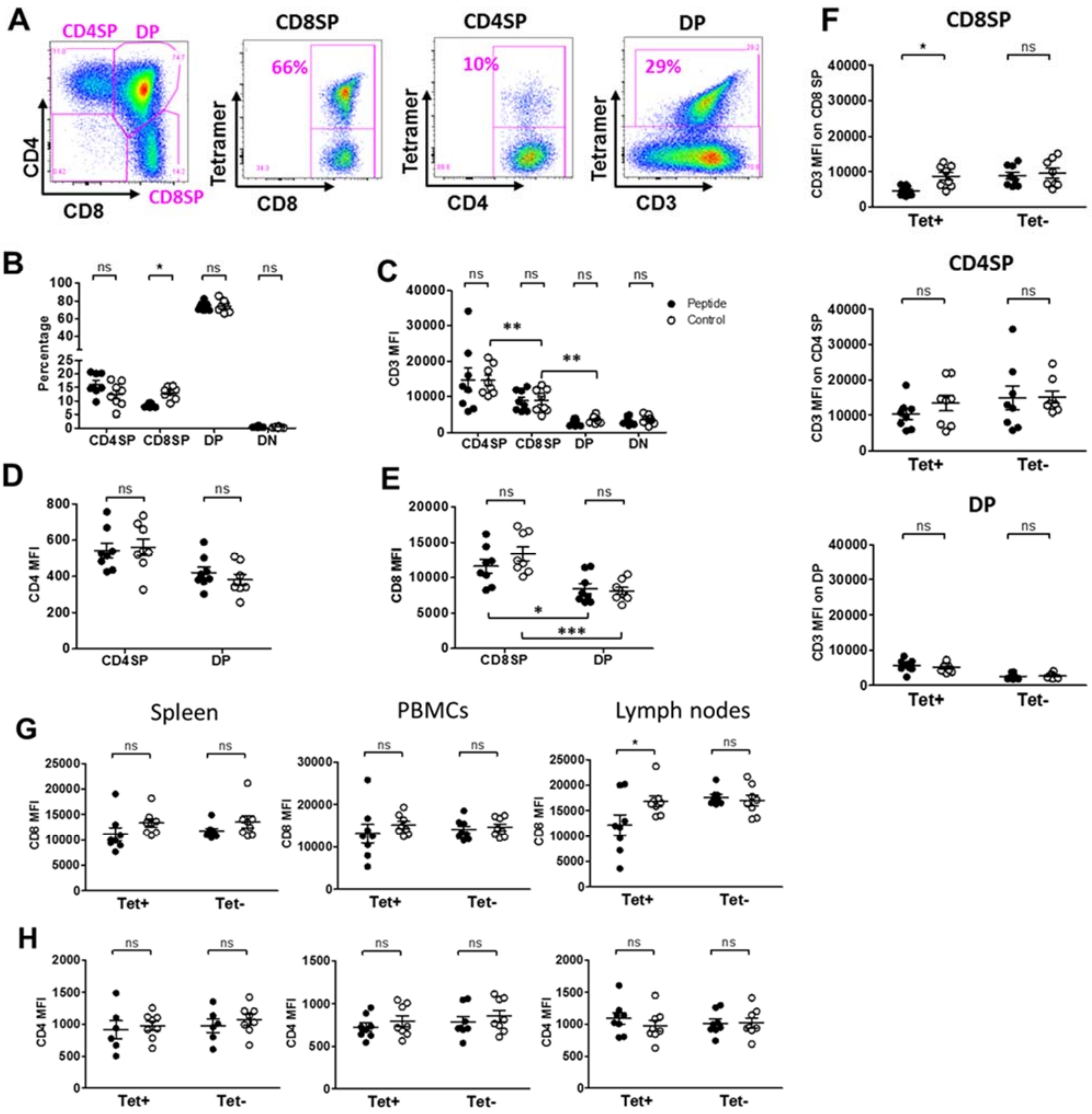

Figure S4: CD3, CD4 and CD8 molecule expression in different tissue. (**A**) Tetramer staining on thymic CD4SP, CD8SP and DP populations, after first gating on live, singlet and $CD45^{+}$ cells. (**B**-**E**) Flow cytometric analysis of total thymocytes for relative frequency of CD4SP, CD8SP, DP and DN populations (**B**), CD3 expression (MFI) on CD4SP, CD8SP, DP and DN populations (**C**), CD4 expression (MFI) on CD4SP and DP cells (**D**) and CD8 expression (MFI) on CD8SP and DP cells (**E**). (**F**) CD3 MFI on Tet^{+} and Tet^{-} cells in CD4SP, CD8SP and DP populations of thymic graft. (**G**) Expression of CD8 on Tet^{+} and Tet- $CD8^{+}$ T cells from spleen, PBMCs and lymph nodes. (**H**) Expression of CD4 on Tet^{+} and Tet^{-} $CD4^{+}$ T cells from spleen, PBMCs and lymph nodes. All graphs show the mean ± SEM, from n=8 mice per group).

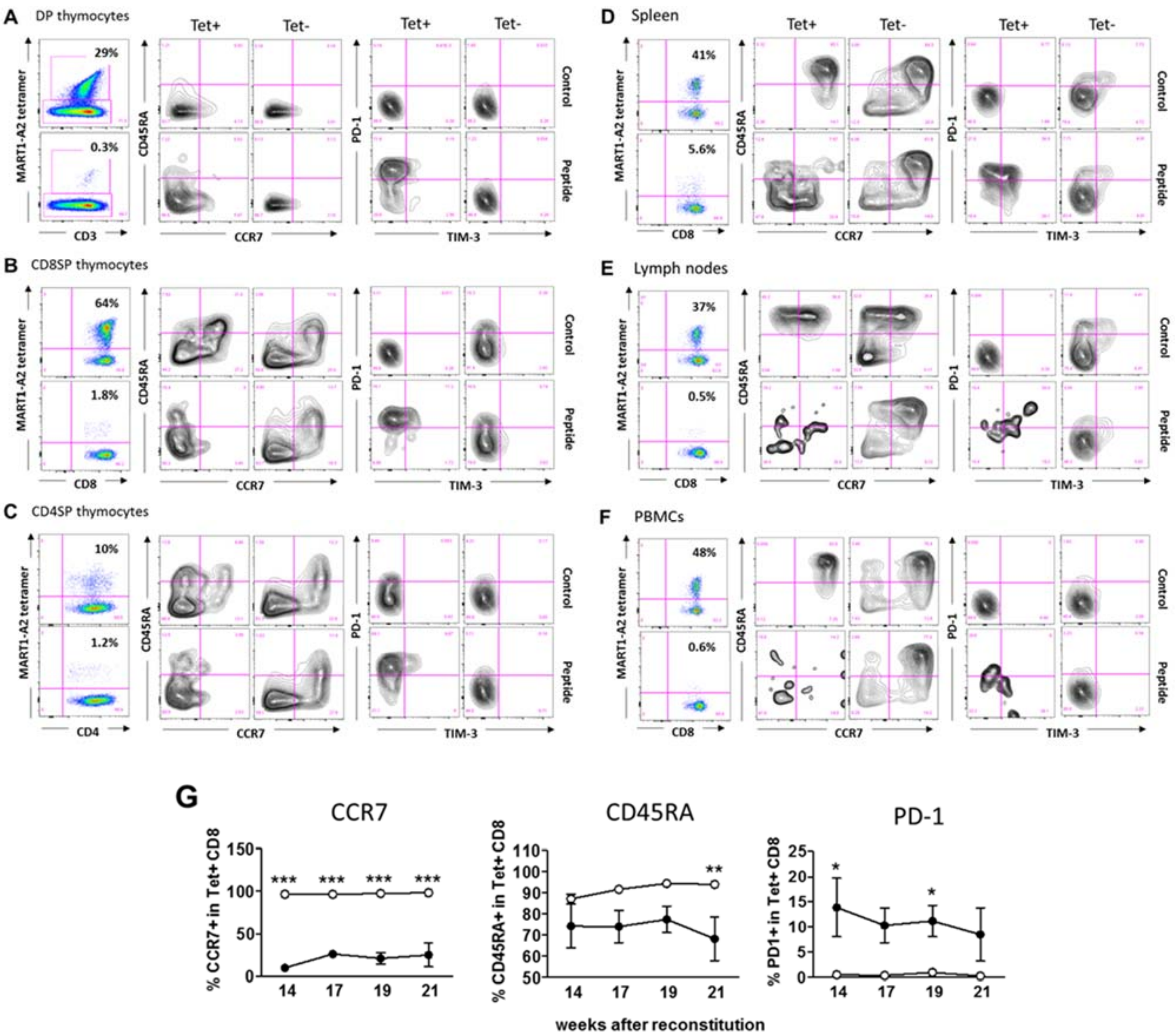

Figure S5: Phenotype of MART1-reactive T cells in the presence or absence of antigen. (**A**-**C**) Representative flow cytometry plots of Tetramer staining among DP (**A**), CD8SP (**B**) and CD4SP (**C**) thymocytes from Control group (upper row) and Peptide group (lower row) at end point, and the expression of CCR7 vs CD45RA and PD-1 vs TIM-3 on Tet^+ and Tet^- cells. (**D**-**F**) Representative flow cytometry plots of Tetramer staining among $CD8^+$ T cells from spleen (**D**), lymph nodes (**E**) and PBMCs (**F**) from Control group (upper row) and Peptide group (lower row) at end point, and the expression of CCR7 vs CD45RA and PD-1 vs TIM-3 on Tet^+ and Tet^- cells. (**G**) Percentage of $CCR7^+$, $CD45RA^+$ and PD-1^+ in Tet^+ cells in the blood of humanized mice at different time points after reconstitution (mean ± SEM, Control group (open circles) n=9; Peptide group (filled circles) n=8).

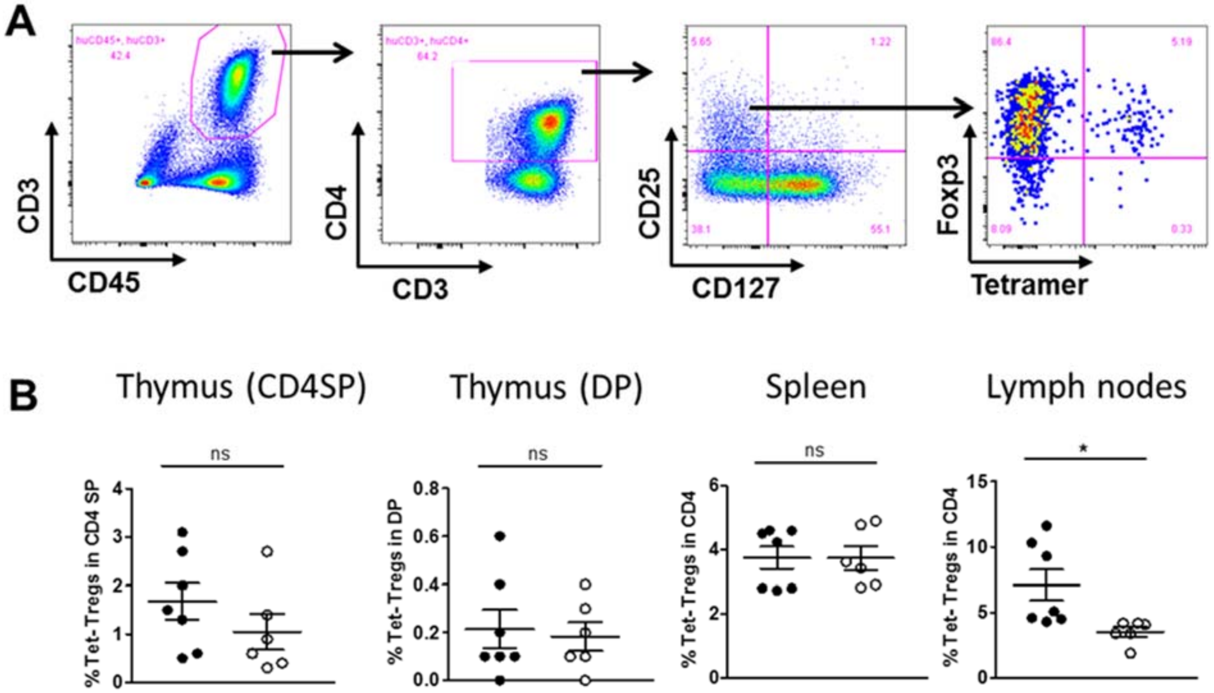

Figure S6: Selection of regulatory T cells. (**A**) Gating strategy for spleen Treg cells, after first gating on live, singlet cells (Control group shown). (**B**) Flow cytometry analysis showed percentage of Tetramer-negative Tregs (Foxp3^{+} CD25^{hi} CD127^{-}) among CD4SP and DP cells in thymic graft, or CD4^{+} T cells in spleen and lymph nodes (mean ± SEM, Control group (open circles) n=6; Peptide group (filled circles) n=7).

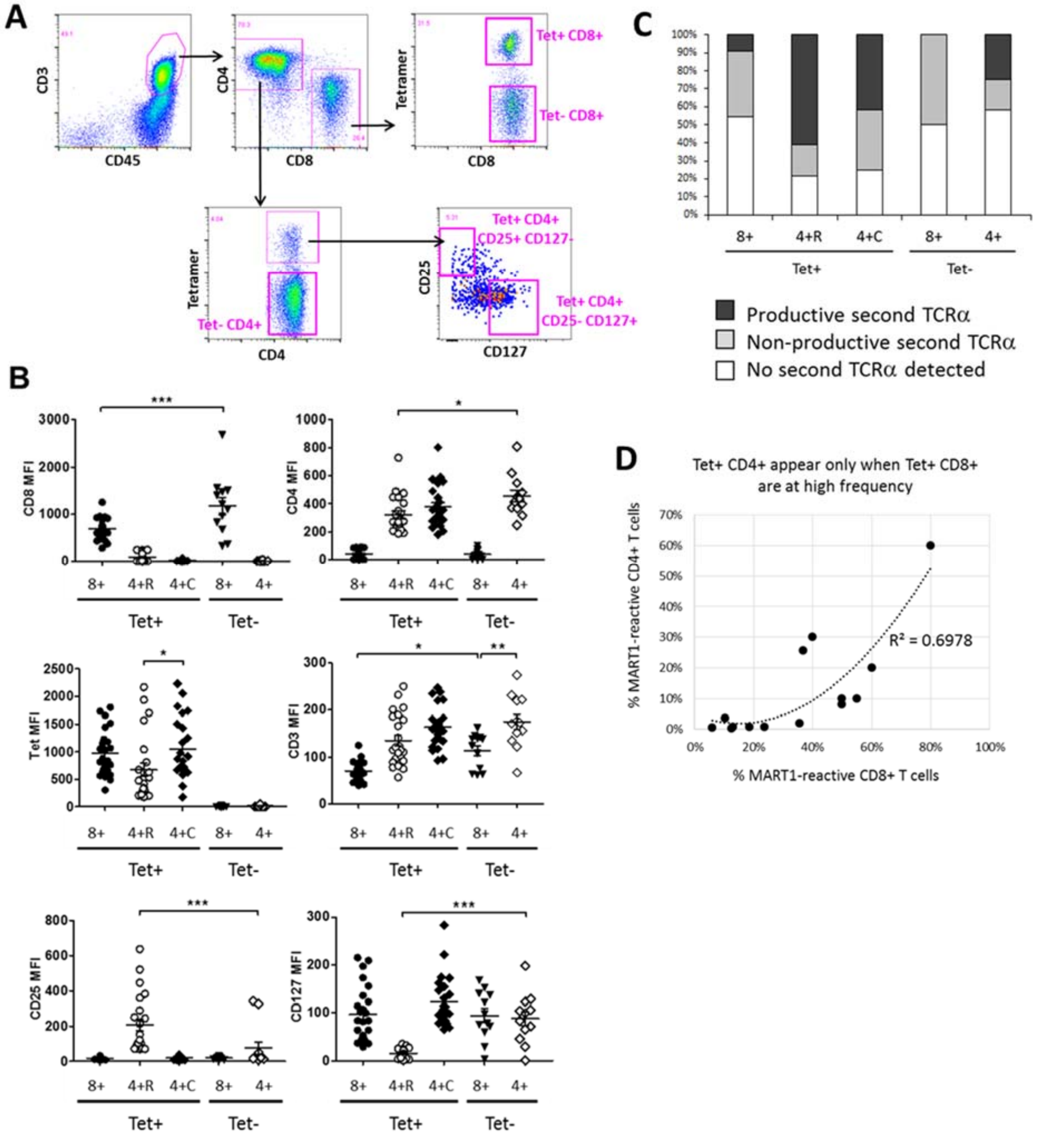

Figure S7: (**A-C**) Single cell sorting: gating strategy and index sorting data. (**A**) Gating strategy for single cells sorting of T cells from splenocytes of a Control group mouse (after gating on live, singlet cells). Cells were sorted into 5 populations of single cells: Tet^{+} CD8^{+} (n=24), Tet^{-} CD8^{+} (n=12), Tet^{+} CD4^{+} CD25^{+} CD127^{-} (n=23), Tet^{+} CD4^{+} CD25^{-} CD127^{+} (n=24) and Tet^{-} CD4^{+} cells (n=12). (**B**) Expression of CD8, CD4, CD3, TCR, CD25 and CD127 (MFI) on single cells in the 5 populations above (mean ± SEM). (**C**) Percentage of clones that express a productive second TCRα, a non-productive second TCRα, or had no second TCRα detected (the last two equivalent to single TCR). Abbreviations: 4+R, CD4^{+} CD25^{+} CD27^{-} (regulatory); 4+C, CD4^{+} CD25^{-} CD127^{+} (conventional). (**D**) Percentage of MART1-reactive CD4^{+} T cells that develop in a humanized mouse based on the percentage of MART1-reactive CD8^{+} T cells, plotted based on Table S1.

Tissue	% Tet+ among CD8+	% Tet+ among CD4+	Study
Thymus (SP) (approx. average)	80%	60%	Giannoni
Spleen (approx. average)	40%	30%	Giannoni
Thymus (SP) (approx. average)	60%	20%	This study
Spleen (approx. average)	50%	8%	This study
Lymph nodes (approx. average)	50%	10%	This study
PBMCs (approx. average)	55%	10%	This study
Thymus (mouse 9)	36.7%	25.5%	Vatakis
Thymus (mouse 6)	35.6%	1.9%	Vatakis
Thymus (mouse 1)	23.8%	0.8%	Vatakis
Thymus (mouse 10)	18.5%	0.7%	Vatakis
Thymus (mouse 3)	13.1%	0.7%	Vatakis
Thymus (mouse 4)	12.5%	0.2%	Vatakis
Thymus (mouse 2)	10.4%	3.6%	Vatakis
Thymus (mouse 5)	6%	0.5%	Vatakis

Table S1: Percentage of MART1-reactive $CD4^+$ T cells that develop in a humanized mouse based on the percentage of MART1-reactive $CD8^+$ T cells.

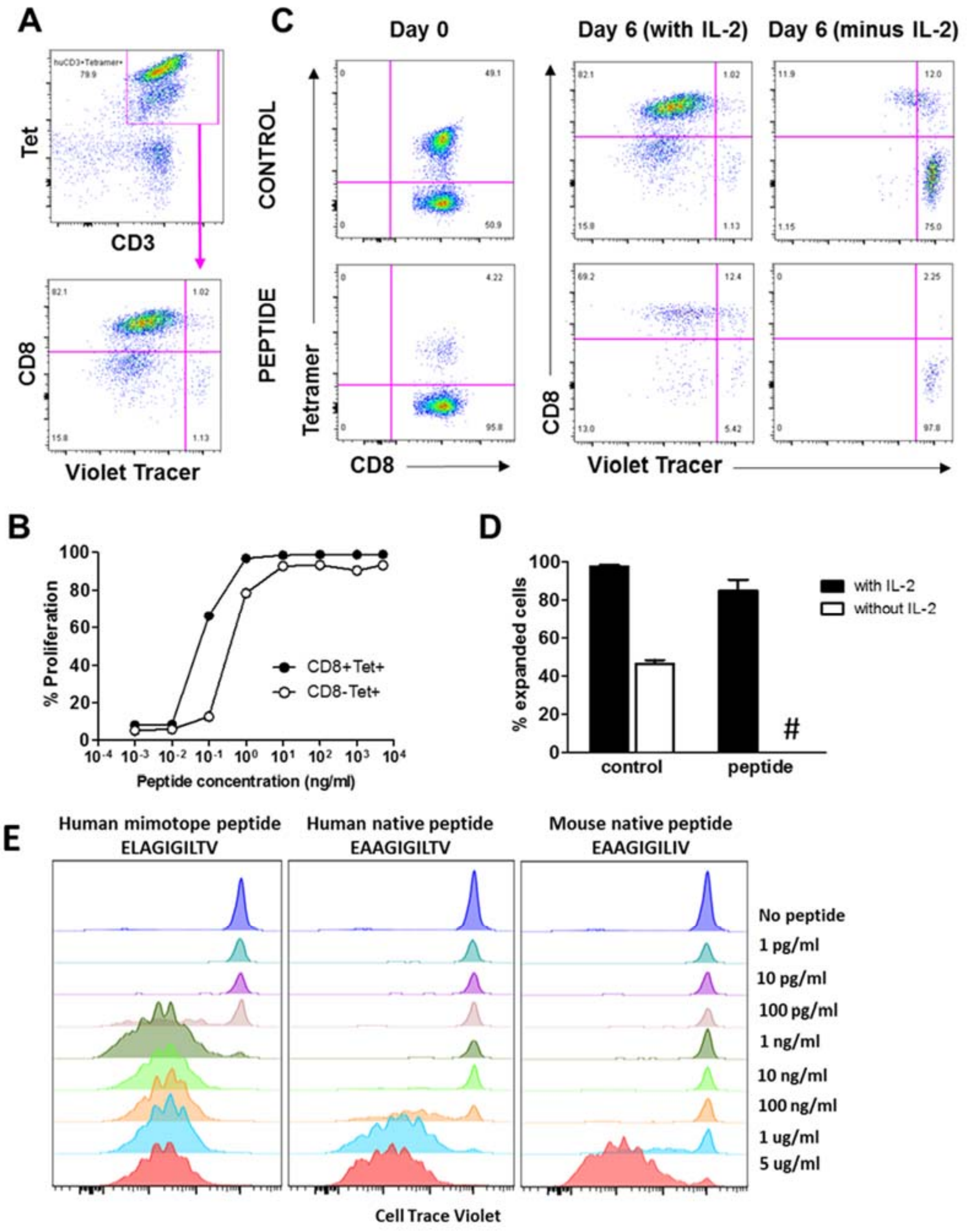

Figure S8: Proliferation of MART1-reactive CD8^{+} and CD8^{-} T cells (gated on CD3^{+} Tet^{+} T cells) 6 days after stimulation of splenocytes from a Control group mouse with 5 µg/ml MART1 peptide and IL-2 (20 U/ml) (**A**) or titrated concentrations of MART1 peptide with 20 U/ml IL-2 (**B**). Representative proliferation of CD8^{+} T cells (gated on CD3^{+} Tet^{+} T cells) 6 days after stimulation of splenocytes from Control and Peptide groups with 5 µg/ml MART1 peptide ± IL-2 (20 U/ml) (**C**) and overall data (from n=3 mice per group) (**D**). # indicates that proliferation could not be determined because there was practically no cell left alive after 6 days. Day 0 data were gated on CD3^{+} T cells. (**E**) **Proliferation of MART1-reactive CD8^{+} T cells to variants of the MART1$_{26\text{-}35}$ peptide.** Splenocytes from the Control group were labeled with Violet Tracer and restimulated in vitro with different concentrations of the human mimotope peptide (the variant expressed in our model), the human native peptide and the mouse native peptide for 7 days. The proliferation of Tet^{+} CD8^{+} T cells is depicted.
